# Development of New Azomethine Metal Chelates Derived from Isatin: DFT and Pharmaceutical Studies

**DOI:** 10.3390/ma16010083

**Published:** 2022-12-22

**Authors:** Abdulrhman A. Al-Shamry, Mai M. Khalaf, Hany M. Abd El-Lateef, Tarek A. Yousef, Gehad G. Mohamed, Kariman M. Kamal El-Deen, Mohamed Gouda, Ahmed M. Abu-Dief

**Affiliations:** 1 Department of Chemistry, College of Science, King Faisal University, Al-Ahsa 31982, Saudi Arabia; 2 Department of Chemistry, Faculty of Science, Sohag University, Sohag 82534, Egypt; 3 Department of Chemistry, Science College, Imam Mohammad Ibn Saud Islamic University (IMSIU), P.O. Box 90950, Riyadh 11623, Saudi Arabia; 4 Toxic and Narcotic Drug, Forensic Medicine Department, Mansoura Laboratory, Medicolegal Organization, Ministry of Justice, Cairo 11435, Egypt; 5 Chemistry Department, Faculty of Science, Cairo University, Giza 12613, Egypt; 6 Nanoscience Department, Basic and Applied Sciences Institute, Egypt-Japan University of Science and Technology, New Borg El Arab 21934, Egypt; 7 Department of Chemistry, College of Science, Taibah University, Medina 42344, Saudi Arabia

**Keywords:** isatin Schiff base ligand, spectroscopic, antimicrobial screening, anticancer activity

## Abstract

Through the condensation of isatin (indoline-2, 3-dione) and aniline in a 1:1 ratio, a Schiff base ligand was synthesized and characterized via (^1^H-NMR, mass, IR, UV-Vis) spectra. Elemental analyses, spectroscopy (^1^H-NMR, mass, UV-Vis), magnetic susceptibility, molar conductivity, mass spectra, scanning electron microscope (SEM), and thermal analysis have all been used to characterize a series of Cr(III), Mn(II), Fe(III), Co(II), Ni(II), Cu(II), Zn(II), and Cd(II) metal complexes derived from the titled ligand. The metal-to-ligand ratio is 1:1, according to the analytical data. The Schiff base ligand displayed bidentate behavior with NO coordination sites when it bonded to metal ions, as seen by the IR spectra. The magnetic moment measurement and UV-Vis spectral investigation showed the octahedral geometry of the Cr(III), Fe(III), Co(II), Ni(II), and Zn(II) complexes, whereas they suggested the tetrahedral geometry of the Mn(II), Cu(II), and Cd(II) complexes. The thermal analysis study confirmed the presence of both hydrated and coordinated water molecules in all the compounds, except for the Mn(II) complex, and showed that the complexes decomposed in three or five decomposition steps leaving the corresponding metal oxide as a residue. The ligand and its metal complexes’ antibacterial efficacy were evaluated. The findings showed that the metal complexes had stronger antibacterial properties than the ligand alone. The ligand and its metal complexes’ anticancer properties were also investigated. A DFT investigation is also reported to gather information regarding the electronic features of the ligand and its metal complexes. Finally, drug-likeness and ADME characteristics were also calculated as parameters.

## 1. Introduction

Schiff bases synthesized from amino and carbonyl compounds are a significant family of ligands that coordinate metal ions via azomethine nitrogen and have been the subject of extensive research [1]. The world of medicine and pharmaceuticals uses a class of chemicals known as Schiff bases extensively. They demonstrate biological functions, such as antibacterial [2,3,4,5,6], antifungal [4,5,6,7,8], and anticancer activities [8,9]. Extraction, enzyme mimics, antibiotics, natural compounds such as marine alkaloids, and pyridine-based Schiff base systems play a significant part in host-guest systems [10,11]. There has been a lot of research conducted on transition metal complexes with Schiff base ligands as antibacterial and anticancer drugs. Metal complexes are used in a wide range of medical, scientific, and industrial processes. They also play a significant role in organic synthesis and catalysis [12,13,14,15].

Isatin (1H-indol-2, 3-dione) is a flexible synthetic substrate that can be utilized to make a wide range of heterocyclic compounds, including indoles and quinolones, as well as providing a starting point for the synthesis of pharmaceuticals. Isatin has also been discovered in mammalian tissues, and its cis-dicarbonyl moiety makes it a potentially useful substrate for the production of metal complexes, either alone or deprotonated. It came about as a result of research into the biological and pharmacological effects of isatin derivatives [16,17,18].

Isatin is a crucial raw material for the synthesis of a diverse range of bioactive chemicals due to its adaptability in synthetic processes. Among other things, isatin compounds have antiviral, anti-inflammatory, anticonvulsant, and anticancer properties. Recent publications [19,20] include studies on the pharmacological properties and synthesis of isatin, including oxindoles and indoles.

Several novel Schiff bases of isatin have been reported with a variety of pharmacological actions, including antimicrobial and antiviral activities [21]. On the other hand, several reports concerned with the synthesis of Schiff base complexes of isatin and their biological activity have been reported [22,23,24].

Thus, the need to prepare new Schiff base complexes of isatin with great biological significance is the propelling force for this research.

With the help of a condensation reaction between isatin, aniline, and its metal complexes and transition metal ions, the author of this study aims to create a Schiff base ligand. Following that, their characterization was investigated using a variety of methods, including elemental analyses, spectroscopy (^1^H-NMR, IR, and UV-Vis), mass, magnetic susceptibility, molar conductivity, and thermal analysis. The molecular stability and bond strengths between the interacting metal ions and the ligand were demonstrated by the density functional theory (DFT). The ability of the Schiff base ligand and its complexes to prevent microbial growth were examined. Additionally, the anticancer activity was examined, and acceptable outcomes were found.

## 2. Employed Experiments for the Study

### 2.1. Starting Materials, Reagents Instruments and Solutions

All chemicals and instruments employed in the preparation and characterization of the compounds under inspection were supplied to the Supporting Information.

In order to prepare the metal complexes at a concentration of 1 × 10^−3^ M and measure the conductivity of each metal complex, a certain weight was dissolved in DMF. The Schiff base ligand solutions and their metal complexes were kept in a refrigerator. By carefully diluting the previously generated stock solutions, solutions of the Schiff base ligand and metal complexes at a concentration of 1 × 10^−4^ M were created to record UV-Vis spectra.

### 2.2. Synthesis of Schiff Base Ligand

A mixture of 1H-indole-2,3-dione (2.718 mmol, 0.4 g) dissolved in 15 mL ethanol and aniline (2.718 mmol, 0.253 g) dissolved in 15 mL ethanol was mixed and refluxed while being stirred for roughly two hours whereupon the Schiff base ligand (L) was created. It underwent filtering, recrystallization from hot ethanol, washing with diethyl ether, and vacuum drying.

### 2.3. Synthesis of Metal Complexes

The suitable metal chloride salt (1.80 mmol, 20 mL) was combined in a 1:1 molar ratio with the Schiff base ligand (0.4 g, 1.80 mmol, 20 mL) in a hot ethanolic solution (60 °C) to create the complexes for Cr(III), Mn(II), Fe(III), Co(II), Ni(II), Cu(II), Zn(II), and Cd(II). The complexes precipitated when the mixture was agitated under refluxing for an hour. They were gathered by filtration and cleaned by repeatedly washing them in diethyl ether. The complexes were found to be air-stable and have high melting points.

### 2.4. DFT Studies

For the ligand, and its metal complexes, geometry optimization calculations and vibrational analysis were first carried out. The software Gaussian 09 was used for all the calculations [25]. Density functional theory (DFT), with the B3LYP functional, 6-31+G (d, p) basis set for the ligand atoms and LANL2DZ for the center metal ions, was used to optimize the ligand and its metal complexes. The B3LYP functional is said to have assisted researchers in achieving satisfactory transition metal complex geometries at low computational costs [26,27]. B3LYP/LANL2DZ+6-31+G (d,p) has previously been used successfully, and its applicability to metal complexes have been clearly stated [28].

### 2.5. Antimicrobial Activity

This study’s methodology was followed exactly as it has been explained previously [29,30]. By using the diffusion agar technique, four bacteria (Gram-positive: *Bacillus Subtilis* and *Staphylococcus aureus* and Gram-negative: *Neisseria gonorrhoeae* and *Escherichia coli*) and two fungi (*Candida albicans* and *Aspergillus fumigatus*) were examined. The reference molecules for the antibacterial and antifungal activity were amikacin and ketoconazole, respectively. Three duplicates of each experiment were utilized in each, and the mean value was used to visualize the results [29,30].

### 2.6. Anticancer Activity

Using the Skehan and Storeng technique [31,32], the compounds’ activity was evaluated. The cell monolayer was treated with various concentrations of the substances under research (0, 5, 12.5, 25, 50, and 100 µg/mL), and triplicate wells were made for each dose. To obtain the survival curve of the breast tumor cell line for each substance, the technique was followed, and the relationship between the survival percentage and medication concentration was shown [31,32].

Calculation:

The formula used to determine the percentage of cell survival was survival fraction = O.D (treated cells)/O:D (control cells).

The amounts of the Schiff base or complexes needed to generate a 50% inhibition of cell growth are known as the IC_50_ values. The experiment was conducted three times.

## 3. Results and Discussion

### 3.1. Characterization of Schiff Base Ligand (L)

The Schiff base (L) was synthesized and put through a series of assays, including elemental, mass, ^1^H-NMR, IR spectral, and thermal. The removal of a single sharp and strong band at 3424 cm^−1^ that corresponds to (NH_2_) in free aniline served as a sign that indoline-2,3-dione and aniline had condensed. In 1728 and 1696 cm^−1^, which correspond to the free isatin drug’s v(C=O) and (NHCO), respectively, two significant absorption bands were seen. The prepared Schiff base ligand’s IR spectra revealed a new band for the azomethine (C=N) stretching vibration at 1616 cm^−1^, along with the disappearance of (NHCO), confirming the ligand’s synthesis. The Schiff base ligand (L) ^1^H’s NMR spectrum revealed several signals between 7.003 and 7.583 ppm, which are attributable to the aromatic protons. The removal of the amino group is visible in the spectra of the metal complex and Schiff base ligand. In typical organic solvents, such as ethanol and DMF, the ligand is soluble. The molecular ion peak (M^2+^) at *m*/*z* = 223.97 amu was seen in the mass spectrum of the free ligand, supporting the findings of the elemental analyses.

### 3.2. Elemental Analyses of Complexes

The complexes formed in a ratio of 1:1 had a composition of [M:L] according to the elemental analysis results (Table 1). The complexes, however, were colored, produced in high purity, and were soluble in DMSO and DMF but were insoluble in water and ethanol as well as methanol and acetone.

### 3.3. IR Spectral Studies

In order to establish the binding mechanism of the Schiff base ligand to the appropriate metal ion, the IR spectrum of the ligand was compared with that of the metal complexes [33,34]. The collected data are listed in Table S1. The v(C=N) of the azomethine group was identified in the free ligand (L), which displayed a strong band at 1616 cm^−1^. Upon complexation, this band’s strength and frequency changed. In every compound, this band changed to a higher frequency, demonstrating the role of (C=N) in the coordination of the metal ions [35]. The (C=O) group was identified by the sharp band displayed in the Schiff base ligand IR at 1728 cm^−1^. As the band changed to higher and lower frequencies, the complexation was verified. This was further supported by the emergence of new bands in all the complexes during the IR spectra in the ranges of 503–529 cm^−1^ and 415–492 cm^−1^ as a result of (M/O) and (M/N) stretching vibrations, respectively [36,37]. The ranges of 872–879 and 911–955 cm^−1^ have seen the appearance of the bands resulting from coordinated water molecules in various complexes of the Schiff base ligand. It can be assumed that the Schiff base ligand behaved as a neutral bidentate ligand and was coupled to the metal ions via oxygen and nitrogen atoms.

### 3.4. Molar Conductivity Measurements

The molar conductivity of a DMF solution (10^−3^ M) was measured at room temperature to determine the ionic nature of the metal complexes. According to the data presented in Table 1, the molar conductivity values for the complexes of Cr(III), Fe(III), Co(II), and Cd(II) were 65.10, 71.50, 63.60, and 83.20 Ω^−1^mol^−1^cm^2^, respectively. These findings revealed that these complexes were electrolytes and ionic in nature, while the molar conductivity of the Mn(II), Ni(II), Cu(II), and Zn(II) complexes was 9.70, 10.50, 8.6, and 12.00 Ω^−1^mol^−1^cm^2^, respectively. It was proposed that these complexes, in which the chloride ions were located inside the coordination sphere, were non-ionic in nature and non-electrolytes [38].

### 3.5. UV–Vis Spectra

The complexes’ electronic absorption spectral data were collected at room temperature in 10^−4^ M DMF solutions. The Schiff base ligand and its metal complexes in DMF had absorption bands between 200 and 700 nm in their UV-Vis spectra. Two bands at 385 and 265 nm, corresponding to the n-π* and π-π* transitions, were visible in the ligand’s spectrum. The complexes’ spectra displayed strong bands in the high energy region between 256 and 294 nm that could be attributed to the π-π* transition. The charge transfer LMCT band can be ascribed to a new band that has been detected at 414 nm [39]. All compounds showed a disappearance of the 385 nm band.

### 3.6. Electronic Spectra and Magnetic Moments

The significance of the magnetic and electronic spectra in defining the geometrical structures of the metal chelates that were previously investigated is explained in this section (Table S2).

Three absorption bands in the region of 17,421, 19,723, and 23,640 cm^−1^, which correspond to the transitions ^4^T_1g_→^4^T_2g_(F), ^4^T_1g_→^4^A_2g_(F), and ^4^T_1g_→^4^T_g_(P), were visible in the reflectance spectrum of the Co(II) complex, indicating an octahedral geometry surrounding the Co(II) ion. The octahedral shape was confirmed by the magnetic moment value, which was 4.81 BM [40].

Three bands at 15,822, 18,465, and 21,008 cm^−1^ in the Mn(II) complex’s diffused reflectance spectrum could be attributed to the ^6^A_1g_ →^4^T_2g_(D), ^4^T_1g_(D)→^6^A_1g,_ and ^4^T_2g_(G)→^6^A_1g_ transitions, respectively. The presence of the Mn(II) complex in the tetrahedral structure was revealed by the magnetic moment value of 5.63 B.M.

The ^3^A_2g_ → ^3^T_2g_, ^3^A_2g_(F)→^3^T_1g_(F), and ^3^A_2g_(F)→^3^T_1g_(P) transitions were visible in the electronic spectral bands of the Ni(II) complex at 14,456, 16,025, and 23,640 cm^−1^, respectively. A high spin octahedral shape was confirmed by the spectrum transition and magnetic moment values of 3.44 B.M. [41].

The hexa-coordinated Cr(III) complex has three spin-allowed transitions: ^4^A_2g_(F)→^4^T_1g_(P), ^4^A_2g_(F)→^4^T_2g_(F), and ^4^A_2g_(F)→^4^T_2g_(F). Three absorption bands were visible in the diffused reflectance spectrum of the Cr(III) complex at 21,834, 19,841, and 17,301 cm^−1^. The chelate’s reported spectrum is reasonably consistent with that described in the literature. The magnetic moment was 4.14 B.M. at room temperature, which is in line with what is predicted for octahedral Cr(III) complexes [33,42,43].

It was discovered from the diffused reflectance spectrum that the Fe(III) chelate displayed a band at 20,661 cm^−1^, which may be attributed to the ^6^A_1g_→T_2g_(G) transition in the complex’s octahedral geometry [44]. At 17,421 and 15,267 cm^−1^, the ^6^A_1g_→^5^T_1g_ transition appeared to be broken into two bands. The Fe(III) complex’s measured magnetic moment was 5.08 BM, which indicates an octahedral shape [44].

Two bands were visible in the Cu(II) complex’s diffused reflectance spectrum at 19,120 and 23,310 cm^−1^, respectively, which correspond to the ^2^B_1g_→^2^E_g,_ and ^2^B_1g_→^2^A_1g_ transitions in a tetrahedral geometry [45]. According to the suggested formulas, the octahedral geometry of the Zn(II) complex was postulated. According to the suggested formula, the tetrahedral geometry of the Cd(II) complex was postulated.

### 3.7. Thermal Studies

Thermogravimetric analysis has been used to examine the thermal behavior of metal complexes from room temperature to 1000 °C in a nitrogen atmosphere. Thermograms were used to calculate the complexes’ weight loss percentages, breakdown phases, temperature ranges, and decomposition products as shown in Figure S6. The results obtained by redrawing the TG curves as the percent mass loss vs. temperature (TG) are shown in Table 2.

The chemical formula [C_14_H_10_N_2_O] of the Schiff base ligand was thermally degraded in three separate processes. The loss of the C_6_H_5_N molecule may be responsible for the first and second stages’ estimated mass loss of 40.19% (calcd. = 40.99%) at the temperature range of 70–365 °C. The third stage took place between 365 and 490 °C, with an estimated mass loss of 59.63% (calcd. = 59.00%), which is associated with the loss of a C_8_H_5_NO fragment.

The thermogram of the Cr(III) complex revealed that the initial weight loss, representing the loss of an H_2_O molecule, occurred between 90 and 165 °C, with a found mass loss of 4.76% (calcd. = 4.13%). The second phase may be attributed to the breakdown of 2H_2_O and C_6_H_6_ molecules with a found mass loss of 26.48% (calculated at 26.85%) within the temperature range of 165–265 °C. The loss of C_8_H_5_N_2_Cl_3_ molecules during the penultimate stage, which took place between 265 and 515 °C and resulted in a mass loss of 56.51% (calculated as 56.29%), is what caused the formation of ½Cr_2_O_3,_ which was tainted with carbon. The overall weight decrease was 87.75% (calculated to be 87.27%).

The Mn(II) complex thermogram revealed four stages of breakdown. The loss of C_8_H_6_N_2_Cl molecules in the first and second phases of breakdown within the temperature range of 295–365 °C, with a maximum temperature at 309 and 352 °C, corresponded to a mass loss of 47.50% (calculated at 47.67%). The third and fourth phases, which ranged in temperature from 365 to 560 °C with maximum temperatures at 496 and 541 °C, correspond to the loss of a C_8_H_4_Cl molecule with a mass loss of 31.90% (calculated to be 32.76%), leaving metal oxide (MnO) as a residue. The overall weight decrease was 79.40% (79.43%) (calculated).

There were four separate thermal decompositions of the Fe(III) complex. The overall weight decrease was 83.32% (83.20% calculated). In the first, coordinated water molecules were lost at a mass loss of 4.18% (calculated at 4.08%) during the temperature range from 60 to 100 °C. With an estimated mass loss of 16.54% (calculated loss of 17.12%) in the second step, which took place between 100 and 380 °C, two H_2_O and two 2HCl molecules were eliminated. Within the temperature range of 380–590 °C, the third and fourth steps had a mass loss of 62.60% (calculated as 62.00%), which corresponded to the loss of C_14_H_9_N_2_Cl_2_ molecules, leaving residues of ½Fe_2_O_3_ which were tainted with carbon.

The Co(II) complex underwent five major stages of degradation throughout the thermal decomposition process. The first stage took place between 50 and 80 °C, with an estimated mass loss of 4.63% (calcd. = 4.00%) which could have been explained by the loss of water molecules. With an estimated mass loss of 12.73% (calculated at 12.00%) in the second stage, which took place between 80 and 230 °C, it may be confidently assumed that three water molecules were lost in this step. The third and fourth phases took place between 230 and 495 °C, with the mass loss of C_8_H_5_N_2_ molecules estimated to be 33.20% (calculated = 33.84%). The penultimate stage took place between 495 and 580 °C, with a mass loss of 35.14% (calculated as 35.20%) that corresponded to the removal of the C_6_HCl_2_ molecule. A total mass loss of 85.70% (calculated as 85.04%) left CoO with carbon contamination as a residue.

The Ni(II) complex showed five stages of breakdown. The complex lost three water molecules in the first breakdown step, which took place in the temperature range from 80 to 120 °C with a maximum of 105 °C, for a mass loss of 12.38% (calculated to be 12.70%). In the second and third stages of decomposition, which took place between 120 and 440 °C with maximum temperatures of 187 and 371 °C, the complex lost its water and C6H5 molecules, resulting in an estimated mass loss of 26.61% (calculated to be 26.77%). The fourth and fifth steps took place between 440 and 590 °C, with the greatest temperatures occurring at 525 and 570 °C, respectively. This corresponded to the elimination of the C_8_H_5_N_2_Cl_2_ molecule with a mass loss of 47.10% (calculated as 47.20%), leaving metal oxide (NiO) as a residue. The overall weight loss amounted to 86.09% (calc. 86.67%).

The Cu(II) metal complex’s TG curve showed three stages of breakdown. One hydrated water molecule and HCl with a mass loss of 14.64% (calcd. = 14.55%) could be responsible for the first and second breakdown processes within the temperature range of 105–435 °C with maximal temperatures at 135 and 347 °C, respectively. The third stage resulted in the loss of the C_14_H_9_N_2_Cl_2_ molecule with a mass loss of 64.00% (calculated as 64.21%), leaving metal oxide (CuO) as a residue [46]. The overall weight decrease was 78.64% (78.76% calculated).

The Zn(II) complex broke down into the following four phases. The first and second steps took place between 70 and 225 °C, and they revealed the loss of two H_2_O molecules with a discovered mass loss of 8.36% (calculated at 7.00%). Within the temperature range of 225–365 °C, the third step demonstrated the loss of H_2_O molecules with a mass loss of 8.36% (calculated as 8.00%). The final step revealed that, throughout the temperature range of 365–660 °C, C_14_H_10_N_2_Cl_2_ molecules were lost with a mass loss of 64.30% (calc. 66.00%), leaving metal oxide (ZnO) as a residue [47]. The overall percentage of weight lost was 81.02% (calculated as 81.00%).

The Cd(II) complex broke down into the following three phases. The first step, representing the loss of two water molecules with a mass loss of 7.80% (calculated = 7.90%), was step one at a temperature of 70 to 165 °C, with a maximum temperature of 126 °C. The loss of C_6_H_5_ and water molecules in the second stage was indicated by a mass loss of 20.67% (calculated as 21.71%) over the temperature range of 165–430 °C, with a maximum loss at 384 °C. The final stage involved the loss of a C_8_H_5_N_2_Cl_2_ molecule with a mass loss of 43.35% (calculated to be 45.00%) at a 430–700 °C temperature range. The remnant from the thermogram was the metal oxide CdO.

### 3.8. SEM Study

**Figure 1:**
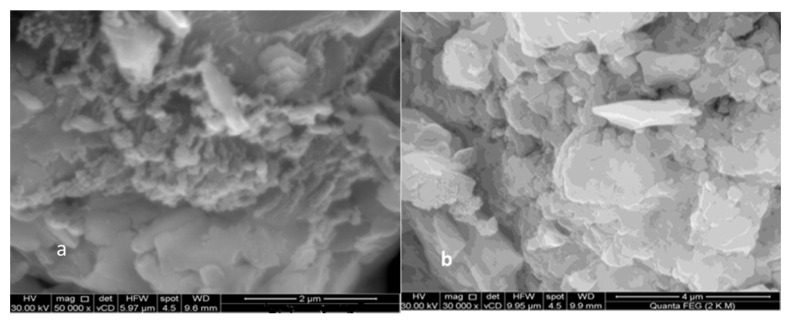
Scanning electron microscope (SEM) of (**a**) Schiff base ligand L and (**b**) Cd(II) complex.

SEM images of the complexes and ligands can be seen in Figure 1. Due to the coordination of the metal ions to the donor sites in the ligand, the SEM micrographs of the Schiff base ligand and Cd(II) chelate were very different from one another [48]. The Schiff base ligand (L) micrograph revealed the existence of well-defined grains with similar morphology, although their sizes were very dissimilar from one another.

The [Cd(L)Cl(H_2_O)]Cl·2H_2_O complex was micrographed, and it showed scattered rods in between the non-uniform platelet formations with varying lateral dimensions. Particles around the size of nanometers were also randomly scattered over these ice rock shape structures, which had an uneven broken ice rock shape morphology. The [Cd(L)Cl(H_2_O)]Cl·2H_2_O complex had an average particle size of 45.00 nm compared to the Schiff base ligand’s 14.57 nm. The compound was crystalline with nanoscale grains, according to the average grain size determined by SEM examination.

**Figure 2:**
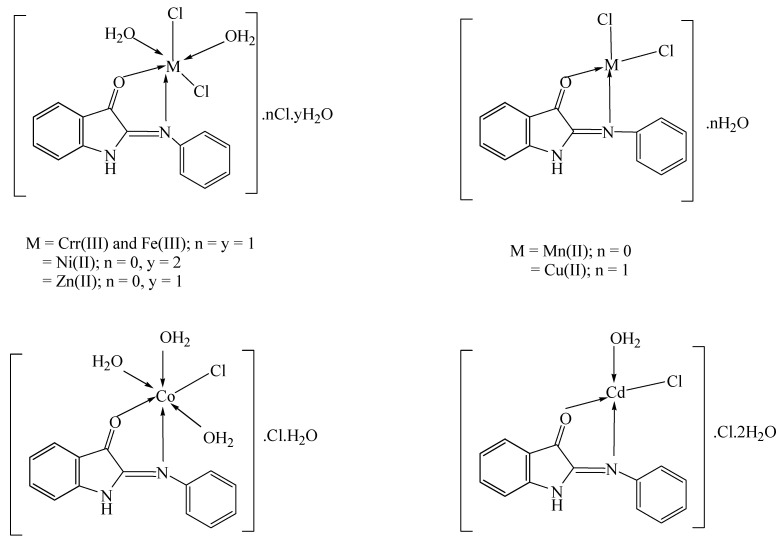
Structure of the investigated metal complexes.

Based on the results of the spectroscopic, elemental, molar conductance, magnetic moment, and thermal analysis data given above, the proposed structures for the metal (II/III) complexes are shown in (Figure 2). According to these findings, the ratio of the ligand to the metal (II/III) ions was 1:1, and the nitrogen and oxygen atoms of the Schiff base ligand served as the metal’s coordination sites.

### 3.9. Calculation of Quantum Chemical Parameters

#### 3.9.1. DFT Calculations Studies

In order to identify some quantum parameters, such as the partial charge of the atoms, the energy molecular orbitals (HOMO and LUMO), and the chemical potential, etc., we incorporated theoretical research using the theorem “DFT” based on computation B3LYP/6-311G (d, p) and LANL2DZ for the center metal ions into the practical portion.

**Figure 3:**
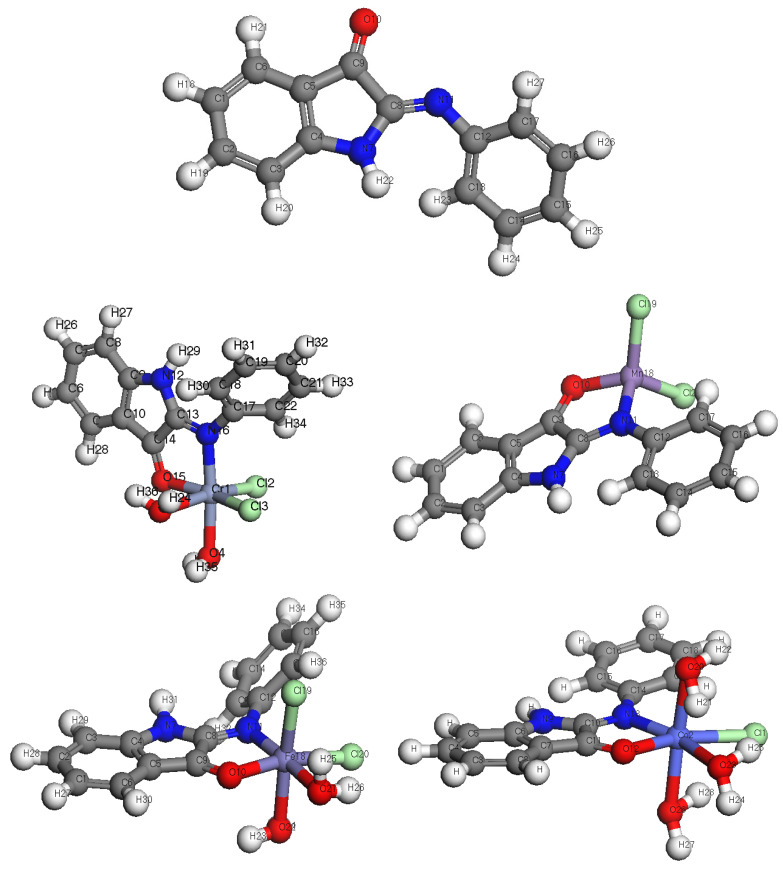

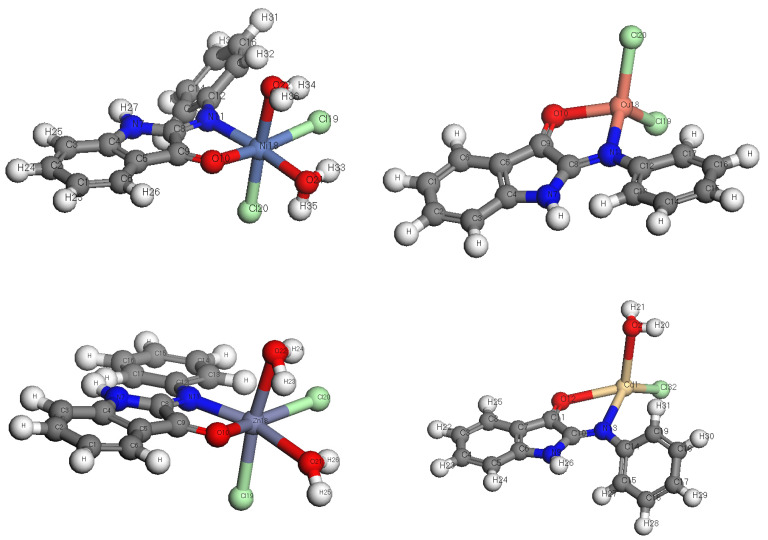
Optimized geometrical structures of L, Cr(III), Mn(II), Fe(III), Co(II), Ni(II), Cu(II), Zn(II), and Cd(II) complexes.

The results of the theoretical DFT calculations (Figure 3) showed different geometrical structures for the free ligand compared to the coordinated metal complexes, which agrees with experimental results. All complexes have an octahedral geometry, while Mn, Cu, and Cd complexes have a tetrahedral geometry in good agreement with the experimental data of electronic spectra and magnetic moment measurements. Table 3 lists the approximate DFT calculations for the properties of the elements ligand, Cr(III), Mn(II), Fe(III), Co(II), Ni(II), Cu(II), Zn(II), and Cd(II), including their electronic energy, heat capacity, entropy (S), thermal energy, polarizability, and dipole moment.

The heat of the formation of the complexes is more negative than that of the ligand; this leads to the ligand stability being less than that of its complexes. It is obvious that more polar molecules dissolve more easily than less polar ones since dipole moments are employed to express a molecule’s polarity. The arrangement is Cu < Mn < Cd < L < Cr < Zn < Co < Ni. Cu is the lowest dipole moment, while Ni is the largest.

Of all the key thermodynamic variables identified for proteins, heat capacity has the most intricate web of ideas and the widest range of consequences for protein folding and binding. Entropy, Gibbs free energy, and enthalpy are given a temperature dependency, changing their signs and determining which one will dominate. The negative values of the free energy and positive values of entropy reveal that complexation reactions are spontaneous processes. The positive values of enthalpy reveal that all formation reactions are endothermic. The order by heat capacity is L < Cu < Mn < Cd < Co < Zn < Fe < Ni < Cr, in which all values are positive and result in the maximum stability and frequent cold denaturation for an unfolding protein [49].

#### 3.9.2. Study of Frontier Orbitals

The energy of the highest occupied molecular orbital (HOMO) and the lowest unoccupied molecular orbital (LUMO), which are related to the ionization potential and electron affinity, defines the electron transfer process as the electron donor or acceptor unit. The gap energy, or ΔE, is the absolute energy difference between the boundary molecular orbitals, which express the reactivity of the compounds. When energy deficits are small, this activity becomes crucial [50].

**Figure 4:**
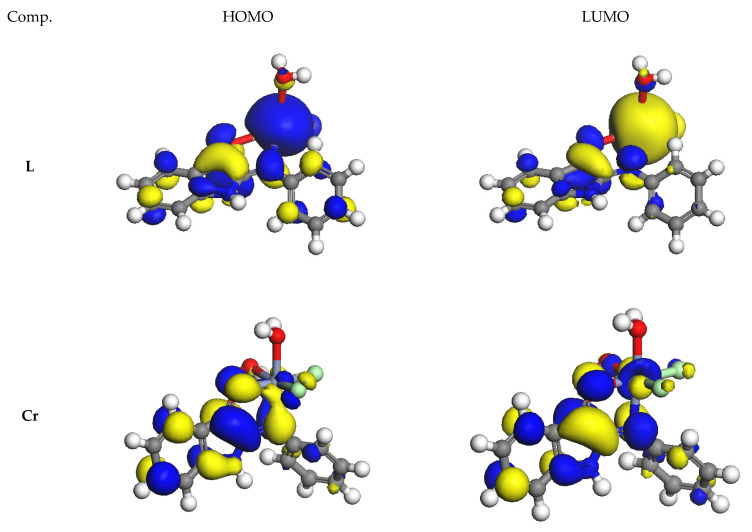

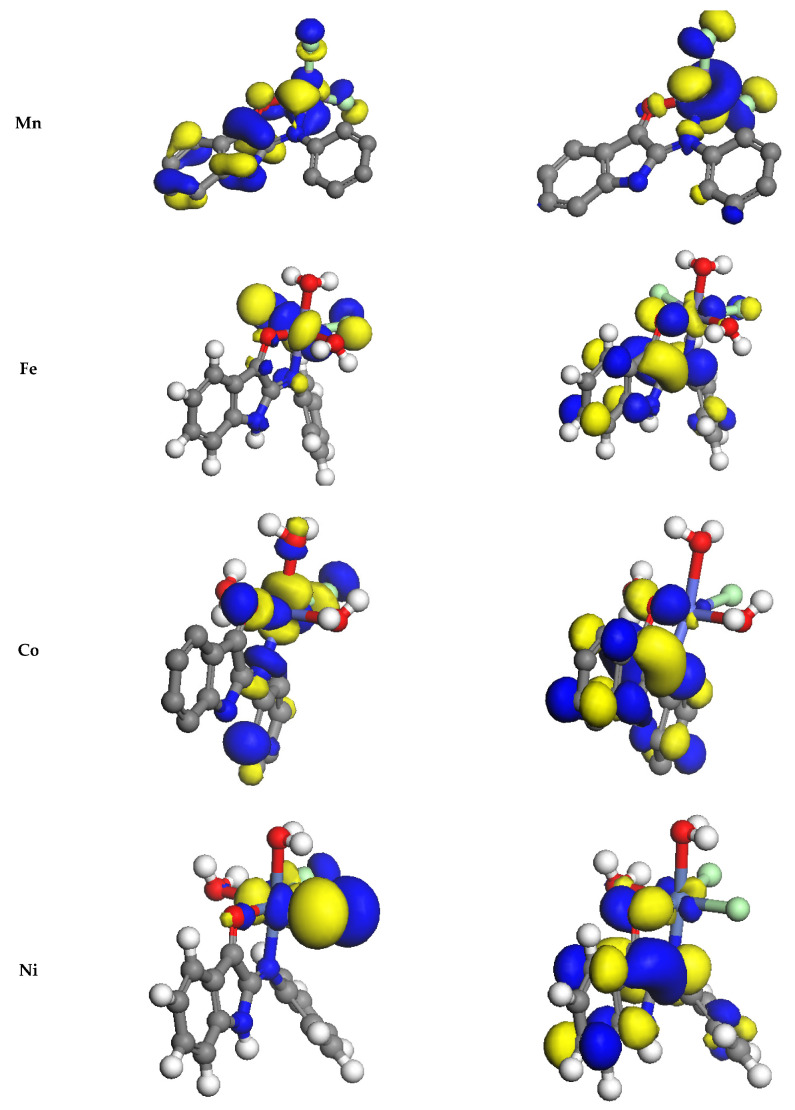

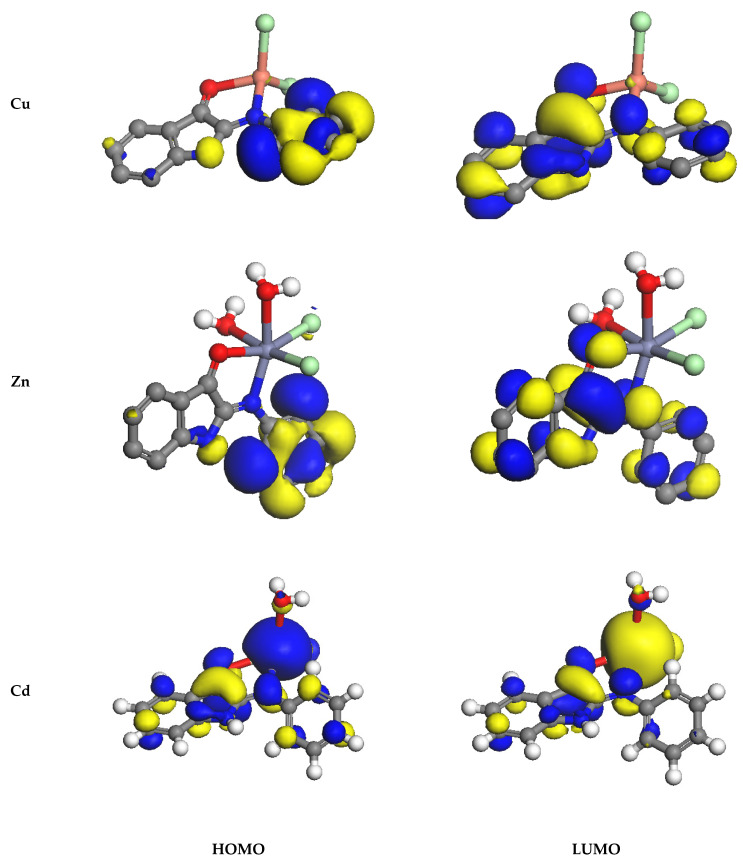
Occupied higher energy and unoccupied lower energy molecular orbitals of Cr(III), Mn(II), Fe(III), Co(II), Ni(II), Cu(II), Zn(II), and Cd(II) complexes.

The charge density distribution of the HOMO and LUMO levels for the studied molecules is shown in Figure 4. HOMOs completely cover the whole molecule, with LUMOs being mainly located throughout the molecular structure of L except for the two phenyl rings. In the case of metal complexes, different locations of HOMO and LUMO are present. The lowest energy gap (ΔE) energy is calculated at 0.14 eV and illustrates the highest reactivity of the Cd complex molecule, which agrees well with the biological experimental data, as the strongest anti-breast cancer activity. Consequently, the energy gap is in the order of Cd < Mn < Co < Fe < Cr < Ni < Zn < Cu < L.

#### 3.9.3. Chemical Reactivity Descriptors

The quantum chemical characteristics of organic compounds can be calculated using terms such as EHOMO and ELUMO. Separation energies (E), absolute electro-negativities (v), chemical potentials (Pi), absolute hardness (g), absolute softness (r), global electrophilicity (x), and global and softness (S) were among the other metrics that were calculated [51,52,53].

Table 4 shows the parameters that were used to evaluate the compounds listed above. The global softness (S), softness (σ), and global hardness (ɳ) parameters affect reactivity and molecular stability. When electrons transfer to an acceptor, soft substances are more reactive than hard ones. The compounds’ ability to draw electrons is measured by their electronegativity (χ), which ranges from 3.72 to 5.42 eV^−1^ [28]. The electrophilicity index (ω) is in the range of 7.68–139.92 eV, which refers to electrophilicity behavior.

### 3.10. In Silico ADME Predictions

An in silico ADME (absorption, distribution, metabolism, and excretion) study of the ligand and its metal complexes were conducted using the Swiss ADME web tool, and critical physiochemical parameters were determined [54,55]. The values of the ligand and its metal complexes’ physiochemical characteristics, lipophilicity, water solubility, pharmacokinetics, and drug similarity are displayed in Tables S3 and S4. With good membrane permeability (BBB) and strong gastrointestinal absorption (GI), all the complexes and the ligand are present (Table 5). The Lipinski rule was followed and is satisfied by all compounds whose pharmacophore or drug-like qualities show that all of their properties are within a tolerable range. Furthermore, as depicted in Figure S7, the oral bioavailability radar chart for the ligand and its metal complexes shows both their anticipated oral bioavailability and their excellent pharmacokinetic results. Additionally, the Radar plot, with the exception of a slight polarity variation, shows a zone with the ideal range of drug similarity [56]. In this in silico ADME prediction, the compounds that met Lipinski’s rule of five requirements without breaking any of them could be good candidates for oral drugs.

### 3.11. Antimicrobial Activity

**Figure 5:**
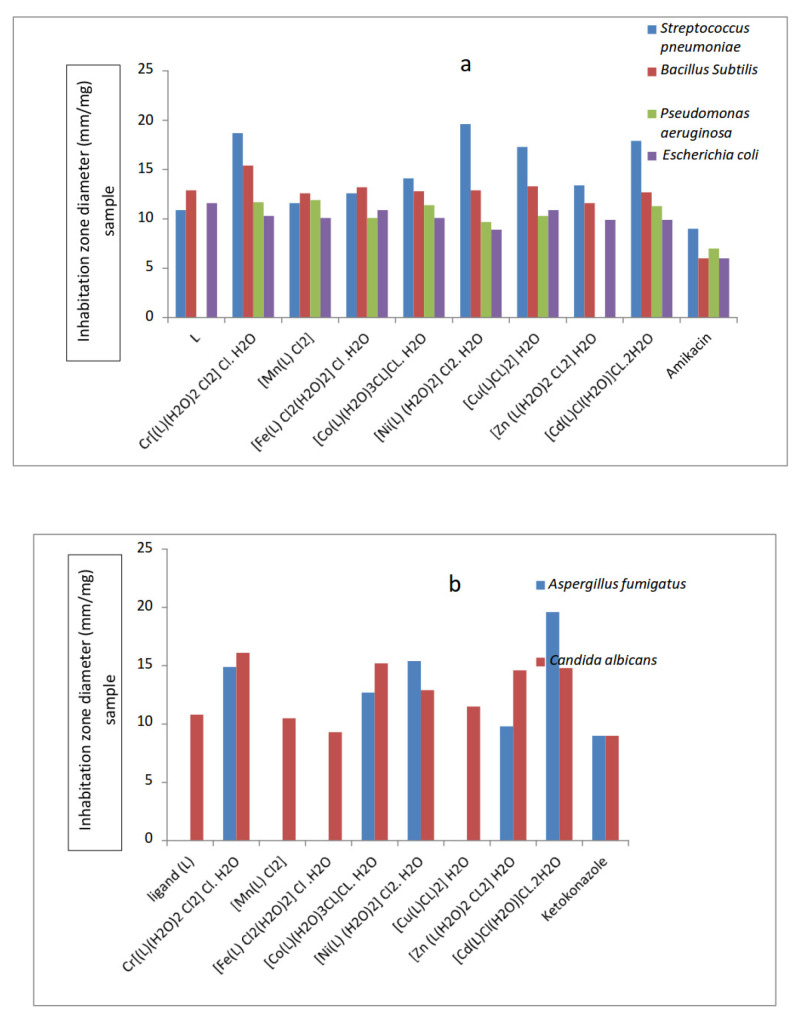
Biological activity of Schiff base ligand and its metal complexes against (**a**) Different bacterial species (**b**) Different fungal species.

A disc diffusion approach was used to evaluate the antibacterial activity of the metal complexes [57]. The five key elements listed below have an impact on how quickly the metal complexes fight bacteria [58,59,60,61]. (i) the effect of the chelating agent, (ii) the kind of coordinated ligands; (iii) their nature; (iv) the type of ion that neutralizes the ionic complex; and (viii) the nuclearity of the metal center in the complex. Complexes with metal ions are better able to enter bacterial cells and are, hence, more efficient at preventing bacterial development [62,63,64]. According to the theory shown in Figure 5, a metal-containing compound that enters a bacterial cell may disrupt proteins and encourage bacterial death.

The higher inhibition zone of the metal complexes compared to those of the ligand can be explained on the basis of Overtone’s idea and chelation theory. The polarity of the metal ion is reduced to a greater extent during chelation because of the overlap of the ligand orbitals and the partial sharing of the metal ion’s positive charge with donor groups. Additionally, it improves the penetration of the complexes into lipid membranes and blocking sites in the microbes’ enzymes, as well as the delocalization of pi-electrons across the entire chelating ring.

The solubility, conductivity, and length of the link between the metal and ligand also play a role in the rise in activity [65]. The Schiff base ligand and its complexes were studied for their potential antibacterial and fungicidal effects. Table S4 and Figure 5 results show that the complexes had more potent inhibitory effects than the parent ligand. *Streptococcus pneumonia, Bacillus subtilis, Pseudomonas aeruginosa*, and *Escherichia coli* were used as test organisms for antibacterial activity in vitro. The following was discovered to be the order of antibacterial activity:

Against *Streptococcus pneumonia*
Ni(II) > Cr(III) > Cd(III) > Cu(II) > Co(II) > Fe(III) > Mn(II)
Against *Bacillus subtilis*
Cr(III) > Cu(II) > Fe(III) > Ni(II) > Cd(III) > Mn(II)
Against *Pseudomonas aeruginosa*
Mn(II) > Cr(III) >Co(II) > Cd(III) > Cu(II) > Fe(III) > Ni(II)
Against *Escherichia coli*
Fe(III) > Cu(II) > Cr(III) > Mn(II) = Co(II) > Zn(II) = Cd(III) > Ni(II)

For fungicidal activity, compounds were screened in vitro against *Aspergillus fumigatus* and *Candida albicans*. The order of antifungal activity was found in the order:

Against *Aspergillus fumigatus*
Cd(III) > Ni(II) > Cr(III) > Co(II) > Zn(II)
Against *Candida albicans*
Cr(III) > Co(II) > Cd(III) >Zn(II) > Ni(II) > Cu(II) > Mn(II) > Fe(III)

By computing the activity index in accordance with the following relation, as shown in Figure S8, the activities of the produced Schiff base ligand and its metal complexes were verified [66,67,68]:

$$Activity\;index\;(A)=\frac{Inhibition\;zone\;of\;compound\;\left(mm\right)}{Inhibition\;zone\;of\;standard\;drug\;\left(mm\right)}\times 100$$

From the data, it was concluded that the Ni(II) complex had the highest activity index while the Mn(II) complex had the lowest activity index.

### 3.12. Anticancer Activity

**Figure 6:**
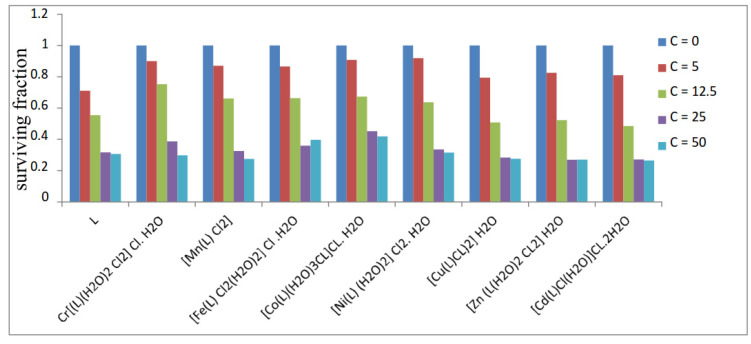
Anticancer activity of Schiff base ligand and its metal complexes.

The newly synthesized Schiff base free ligand and its metal complexes were studied for their cytotoxicity using the breast cancer cell line MCF7. Cell viability is shown in Figure 6 in relation to the changing chemical concentrations. Table S5 summarizes the concentrations (IC_50_) at which half of the maximum effect was observed based on the numerical data. It was discovered that the free Schiff base ligand and metal complexes had anti-breast cancer action. The Co(II) complex was shown to have the lowest activity, with an IC_50_ of 22.30 µg mL^−1^, whereas the Cd(II) complex, with an IC_50_ of 12.00 µg mL^−1^, had the strongest anti-breast cancer activity. These findings offered an excellent illustration of how modifications to the molecular structure of chelation could result in significant variations in anticancer efficacy [69,70,71]. Metal complexes were consequently thought to be a possible anticancer drug and a candidate for additional in vitro and/or in vivo screening stages. We performed the activity of the prepared compounds against the normal cell line (MCF 10 cell line), and the compounds were found to be safer compared with their effect on the cancer cell line. Additionally, we compared the results of anti-cancer activity for the prepared compounds with the standard vinblastine drug and found is comparable (cf. Table S6)

Our study shows that some metal complexes may be more active than the parent Schiff base ligand and may be helpful in the design of new drugs.

## 4. Conclusions

The structure of the newly synthesized Schiff base ligand and its complexes with Cr(III), Mn(II), Fe(III), Co(II), Ni(II), Cu(II), Zn(II), and Cd(II) was confirmed by elemental analyses, spectroscopic research (IR, UV-Vis, ^1^H-NMR) mass spectrometry, magnetic susceptibility, conductivity measurements, and thermogravimetric analysis (TG-DTG). As the ligand is a bidentate donating ligand, the infrared spectra discussed the chelation pathway through the oxygen and nitrogen atoms. Except for the Mn(II), Ni(II), Cu(II), and Zn(II) complexes, which were non-electrolytes, all the complexes were electrolytes according to the molar conductivity data in the DMF solvent. The magnetic measurements show the octahedral geometry of the Fe(III), Co(II), Ni(II), Cr(III), and Zn(II) complexes and the tetrahedral geometry of the Cu(III), Mn(II) and Cd(II) complexes. The free ligand and metal complexes were generated in the nanostructure, according to the SEM studies. At the B3LYP/6-311G(d,p) level involved in the Gaussian 09 program, DFT calculations were performed to examine the optimized structures of the ligands and their complexes. All chemicals were anticipated to be non-AMES hazardous and non-carcinogens with moderate human intestinal absorption (HIA) and Caco-2 permeability, according to ADMET analysis. The antimicrobial test indicated that all the metal complexes significantly outperformed the free Schiff base ligand in terms of antibacterial effectiveness, and some metal complexes exhibited inhibitory activity against breast cancer (MCF7 cell line), with the Cd(II) complex exhibiting the highest anticancer activity and demonstrating the lowest IC_50_ values (12.00 µg mL^−^^1^).

## Figures and Tables

**Table 1:** Schiff base ligand and its metal complex analytical and physical data.

Compound	Colour (%Yield)	m.p. (°C)	% Found (Calcd.)				μ_eff_. (B.M.)	Λm Ω^−1^mol^−1^ cm^2^
			C	H	N	M		
L	Yellow	190	75.45 (75.66)	4.49 (4.54)	12.67 (12.60)	-	-	-
Cr[(L)(H_2_O)_2_Cl_2_]Cl·H_2_O	Green 86	>300	38.65 (38.72)	3.48 (3.54)	5.84 (6.00)	10.94 (11.00)	4.14	60.10
[Mn(L)Cl_2_]	Dark brown 85	>300	48.23 (48.25)	3.27 (3.40)	7.60 (7.90)	14.76 (15.00)	5.63	9.70
[Fe(L)Cl_2_(H_2_O)_2_]Cl·H_2_O	Yellowish brown 80	>300	38.50 (38.90)	3.92 (4.03)	6.18 (6.26)	12.12 (12.17)	5.08	66.50
[Co(L)(H_2_O)_3_Cl]Cl·H_2_O	Faint pink 90	>300	39.09 (39.20)	3.64 (3.70)	5.89 (6.00)	13.10 (13.50)	4.81	58.60
[Ni(L)(H_2_O)_2_Cl_2_]·2H_2_O	Light green 84	>300	39.10 (39.33)	4.04 (4.10)	6.09 (6.19)	12.90 (13.00)	3.44	10.50
[Cu(L)Cl_2_]H_2_O	Brown 83	>300	47.09 (47.60)	3.60 (3.80)	7.17 (7.25)	16.04 (16.15)	1.88	8.60
[Zn(L)(H_2_O)_2_Cl_2_]H_2_O	Yellowish white 87	>300	40.32 (40.55)	3.27 (3.50)	6.18 (6.24)	15.02 (15.25)	Dia.	12.00
[Cd(L)Cl(H_2_O)]Cl·2H_2_O	Brown 86	>300	33.19 (33.50)	2.92 (3.00)	4.94 (5.00)	22.17 (22.50)	Dia.	78.20

**Table 2:** Thermoanalytical data of the metal complexes of the Schiff base ligand.

Complex	TG Range (°C)	DTGmax (°C)	n	Mass Loss Total Mass Loss Estim (Calcd) %	Assignment	Residues
L	70–365 365–490		2 1	40.19 (40.99) 59.63(59.00) 99.82(99.99)	- Loss of C_6_H_5_N. - Loss of C_8_H_5_NO.	-
[Cr(L)(H_2_O)_2_Cl_2_]Cl·H_2_O	90–165 165–265 265–515	102 232 471	1 1 1	4.76 (4.13) 26.48 (26.85) 56.51 (56.29) 87.75 (87.27)	- Loss of H_2_O. - Loss of 2H_2_O and C_6_H_6_. - Loss of C_8_H_5_Cl_3_N_2_	½ Cr_2_O_3_
[Mn(L)Cl_2_]	295–365 365–560	309, 352 496, 541	2 2	47.50 (46.67) 31.90 (32.76) 79.40 (79.43)	- Loss of C_8_H_6_ClN_2_. - Loss of C_6_H_4_Cl.	MnO
[Fe(L)Cl_2_(H_2_O)_2_]Cl·H_2_O	60–100 100–380 380–590	85 284 543, 532	1 1 2	4.18 (4.08) 16.54 (17.12) 62.60 (62.00) 83.32 (83.20)	- Loss of H_2_O. - Loss of 2H_2_O and 2HCl. - Loss of C_14_H_9_Cl_2_N_2_.	½ Fe_2_O_3_
[Co(L)(H_2_O)_3_Cl]Cl·H_2_O	50–80 80–230 230–495 495–580	74 204 289, 445 549	1 1 2 1	4.63 (4.00) 12.73 (12.00) 33.20 (33.84) 35.14(35.20) 85.70 (85.04)	- Loss of H_2_O. - Loss of 3H2O. - Loss of C_8_H_5_N_2_. - Loss of C_6_H_5_Cl_2_.	CoO
[Ni(L)(H_2_O)_2_Cl_2_]2H_2_O	80–120 120–440 440–590	105 187, 371 525, 570	1 2 2	12.38 (12.70) 26.61 (26.77) 47.10 (47.20) 86.09 (86.67)	- Loss of 3H_2_O. - Loss of H_2_O and C_6_H_5_. - Loss of C_8_H_5_N_2_Cl_2_.	NiO
[Cu(L)(Cl)_2_]H_2_O	105–435 435–740	135, 347 673	2 1	14.64 (14.55) 64.00 (64.21) 78.64(78.76)	- Loss of H_2_O and HCl - Loss of C_14_H_9_Cl_2_N_2_.	CuO
[Zn(L)(H_2_O)_2_Cl_2_] H_2_O	70–225 225–365 365–660	106, 197 321 606	2 1 1	8.36 (7.00) 8.36(8.00) 64.30 (66.00) 81.02 (81.00)	- Loss of 2H_2_O. - Loss of H_2_O. - Loss of C_14_H_10_N_2_Cl_2_.	ZnO
[Cd(L)(Cl)(H_2_O)]Cl·2H_2_O	70–165 165–430 430–700	126 384 650	1 1 1	7.80 (7.90) 20.67(21.71) 43.35(45.00) 71.82(71.61)	- Loss of 2H_2_O. - Loss of H_2_O and C_6_H_5_. - Loss of C_8_H_5_N_2_Cl_2_.	CdO

**Table 3:** Total energy (kcal/mol), binding energy (kcal/mol), heat capacity (Cp), entropy (S) (cal/mol-kelvin), enthalpy, free energy (kcal/mol), and dipole moment (Debye) of ligand, Cr(III), Mn(II), Fe(III), Co(II), Ni(II), Cu(II), Zn(II), and Cd(II).

Parameter	L	Cr	Mn	Fe	Co	Ni	Cu	Zn	Cd
Total Energy	−454,624	−4,991,837	−4,623,610	−1,218,134	−988,478	−1,249,679	−1,168,178	−1,284,154	−848,041
Binding Energy	−3315.80	−3982.04	−2992.27	−3978.27	−3676.29	−3996.55	−2903.37	−3324.26	−3803.25
Total Dipole Moment	5.35	6.51	1.09	7.45	8.48	8.89	0.72	7.75	4.66
Enthalpy	8.00	14.56	11.59	13.79	14.96	14.69	10.66	16.04	12.65
Free Energy	−23.89	−31.92	−30.09	−30.55	-32.68	−32.32	−27.99	−37.69	−31.02
Heat Capacity (Cp)	51.61	86.12	63.84	83.53	82.25	85.92	60.61	82.72	74.16
Entropy (S)	106.99	155.91	139.82	148.72	159.78	157.67	129.66	180.18	146.49

**Table 4:** Calculated EHOMO (EH), ELUMO (EL), energy band gap (E_H_–E_L_), chemical potential (μ), electronegativity (χ), global hardness (η), global softness (S), global electrophilicity index (ω), and softness (σ) for L, Cr(III), Mn(II), Fe(III), Co(II), Ni(II), Cu(II), Zn(II), and Cd(II)complexes.

Comp.	EH/eV	EL eV	(EL-EH)/Ev	χ/eV	μ/eV	η/eV	S/eV^−1^	ω/eV	σ/eV^−1^
**L**	−2.9	−4.86	1.96	3.88	−3.88	0.98	1.02	7.68	0.49
**Cr**	−3.41	−4.06	0.65	3.74	−3.74	0.33	3.08	21.46	0.16
**Mn**	−5.19	−5.39	0.2	5.29	−5.29	0.1	10.0	139.92	0.05
**Fe**	−3.69	−4.18	0.49	3.94	−3.94	0.25	4.08	31.60	0.12
**Co**	−4.22	−5.48	1.26	4.85	−4.85	0.63	1.58	18.66	0.32
**Ni**	−3.91	−4.65	0.74	4.28	−4.28	0.37	2.70	24.75	0.19
**Cu**	−4.88	−5.95	1.07	5.42	−5.42	0.54	1.86	27.40	0.27
**Zn**	−4.46	−5.42	0.96	4.94	−4.94	0.48	2.08	25.42	0.24
**Cd**	−3.65	−3.79	0.14	3.72	−3.72	0.07	14.28	98.84	0.04

**Table 5:** Pharmacokinetics and drug likeness of synthesized compounds.

	L	Cr	Mn	Fe	Co	Ni	Cu	Zn	Cd
GI absorption	High	High	High	High	High	High	High	High	High
BBB permeant	Yes	Yes	Yes	Yes	Yes	Yes	Yes	Yes	Yes
P-gp substrate	No	Yes	Yes	Yes	Yes	Yes	Yes	Yes	Yes
CYP1A2 inhibitor	Yes	Yes	Yes	Yes	Yes	Yes	Yes	Yes	Yes
CYP2C19 inhibitor	No	No	No	No	No	No	No	No	No
CYP2C9 inhibitor	No	No	No	No	No	No	No	No	No
CYP2D6 inhibitor	No	Yes	Yes	Yes	Yes	Yes	Yes	Yes	Yes
CYP3A4 inhibitor	No	Yes	No	Yes	No	No	No	Yes	No
Log Kp (skin permeation)	−5.76 cm/s	−6.50 cm/s	−5.62 cm/s	−6.52 cm/s	−5.67 cm/s	−6.54 cm/s	−5.67 cm/s	−6.58 cm/s	−6.76 cm/s
Lipinski	Yes; 0 violation	Yes; 0 violation	Yes; 0 violation	Yes; 0 violation	Yes; 0 violation	Yes; 0 violation	Yes; 0 violation	Yes; 0 violation	Yes; 0 violation
Ghose	Yes	Yes	Yes	Yes	Yes	Yes	Yes	Yes	Yes
Veber	Yes	Yes	Yes	Yes	Yes	Yes	Yes	Yes	Yes
Egan	Yes	Yes	Yes	Yes	Yes	Yes	Yes	Yes	Yes
Muegge	Yes	Yes	Yes	Yes	Yes	Yes	Yes	Yes	Yes
Bioavailability Score	0.55	0.55	0.55	0.55	0.55	0.55	0.55	0.55	0.55

## Data Availability

The raw/processed data generated in this work are available upon request from the corresponding author.

## Supplementary Materials

The following supporting information can be downloaded at: https://www.mdpi.com/article/10.3390/ma16010083/s1, Figure S1: Structure of the Schiff base ligand (L); Figure S2: 1H NMR spectrum of Schiff base ligand (L); Figure S3: Mass spectrum of Schiff base ligand (L); Figure S4: IR spectra of (a) Schiff base ligand (L), (b) Cr(III), (c) Mn(II), (d) Fe(III), (e) Co(II), (f) Ni(II), (g) Cu(II), (h) Zn(II), and (i) Cd(II) complexes; Figure S5: The UV-Vis absorption spectra of (l) Schiff base ligand (L), (a) Cr(III), (b) Mn(II), (c) Fe(III), (d) Co(II), (e) Ni(II), (f) Cu(II), (g) Zn(II), and (h) Cd(II) complexes; Figure S6: Thermal analysis (TG and DTG) of (a) Schiff base ligand (L), (b) Cr(III), (c) Mn(II), (d) Fe(III), (e) Co(II), (f) Ni(II), (g) Cu(II), (h) Zn(II), and (i) Cd(II) complexes; Figure S7: Bioavailability radar chart of the ligand and its metal complexes; Figure S8: Activity index of the prepared Schiff base complexes; Table S1: IR spectra (4000–400 cm^−1^) of Schiff base ligand and its binary metal complexes; Table S2: Diffused reflectance spectral data of binary metal complexes; Table S3: Physiochemical properties of synthesized compounds; Table S4: Lipophilicity and water solubility of synthesized compounds; Table S5: Biological activity of Schiff base ligand and its metal complexes with Gram positive and Gram negative bacteria and with fungi; Table S6: Anti-breast cancer activity of Schiff base ligand and its metal complexes.
