# Supplementary material for: Development of New Azomethine Metal Chelates Derived from Isatin: DFT and Pharmaceutical Studies
Source: Materials (Basel). 2022 Dec 22;16(1):83. doi: 10.3390/ma16010083 (PMC9821024; doi:10.3390/ma16010083)
Supplement: Supplementary file 1 [file materials-16-00083-s001.zip › materials-2046251-supplementary.pdf]

Development of New Azomethine Metal Chelates Derived from Isatin: DFT and Pharmaceutical Studies

Abdulrhman A. Al-Shamry [1], Mai M Khalaf [1,2], Hany M Abd El-Lateef [1,2], Tarek A. Yousef [3,4], Gehad G. Mohamed [5,6], Kariman M. Kamal El-Deen [5] Mohamed Gouda [1] and Ahmed M. Abu-Dief [2,7]

[1]Department of Chemistry, College of Science, King Faisal University, Al-Ahsa 31982, Saudi Arabia
[2]Department of Chemistry, Faculty of Science, Sohag University, Sohag 82534, Egypt
[3] Department of Chemistry, Science College, Imam Mohammad Ibn Saud Islamic University, (IMSIU), Riyadh, KSA, P.O. Box 90950, Riyadh 11623, Saudi Arabia
[4]Toxic and Narcotic drug, Forensic Medicine Department, Mansoura Laboratory, Medicolegal organization, Ministry of Justice, Cairo 11435, Egypt
[5] Chemistry Department, Faculty of Science, Cairo University, Giza 12613, Egypt
[6] Nanoscience Department, Basic and Applied Sciences Institute, Egypt-Japan University of Science and Technology, New Borg El Arab 21934, Egypt
[7]Chemistry Department, College of Science, Taibah University, Medina 42344, Saudi Arabia

S2. Employed experiments for the study

S2.1. Material, reagents, and instrumentation

All the chemicals that were used for the analytical reagent grade (AR) were of the highest purity available. They included the isatin drug (Riesdel-De Haën), aniline (El Nasr Pharmaceutical Chemicals), $CrCl_3$-$6H_2O$ and $MnCl_2$-$2H_2O$ (Sigma), $NiCl_2.6H_2O$, $CoCl_2.6H_2O$, $CuCl_2.2H_2O$, $CdCl_2.2H_2O$ and $ZnCl_2.2H_2O$ (BDH), and $FeCl_3.6H_2O$ (Prolabo). The organic solvents used were ethyl alcohol (90%), diethyl ether, and dimethylformamide (DMF). De-ionized water was usually used in all preparations.

The microanalyses of carbon, hydrogen, and nitrogen were carried out at the Microanalytical Center, Cairo University, Egypt, using CHNS-932 (LECO) Vario Elemental Analyzer. The analyses of the metals followed the dissolution of the solid complex in concentrated HNO_3, the neutralization of the diluted aqueous solutions with ammonia, and the titrating of the metal solutions with EDTA. FT-IR spectra were recorded on a Perkin-Elmer 1650 spectrometer (4000–400 cm^{-1}) in KBr discs. ^1H NMR spectra, as a solution in DMSO-d_6, were recorded on a 300 MHz Varian-Oxford Mercury at room temperature using TMS as an internal standard. Molar conductivities of 10^{-3} M solutions of the solid complexes in the DMF solvent were measured using a Jenway 4010 conductivity meter. The thermogravimetric analyses (TG and DTG) of the solid complexes were carried out from room temperature to 1000 °C using a Shimadzu TG-50H thermal analyzer. The anticancer activity was performed at the National Cancer Institute, Cancer Biology Department, Pharmacology Department, at Cairo University. The optical density (O.D.) of each well was measured spectrophotometrically at 564 nm with an ELIZA microplate reader (Meter tech. Σ 960, USA). The scanning electron microscopic (SEM) image of the complexes was recorded using SEM Model Quanta 250 FEG (Field Emission Gun) attached to the EDX Unit (Energy Dispersive X-ray Analyses), with an accelerating voltage of 30 K.V., magnification 14x up to 1000000, and resolution for Gun.1n, National Research Center, Egypt.

Table S1. IR spectra (4000-400 cm^{-1}) of Schiff base ligand and its binary metal complexes.

**HL**	[Cr(L) $(H_2O)_2Cl_2$] $Cl.H_2O$	[Mn(L)Cl_2]	[Fe(L) $(H_2O)_2$ Cl_2]Cl H_2O	[Co(L) $(H_2O)_3$Cl]Cl. H_2O	[Ni(L)$(H_2O)_2$ Cl_2].2H_2O	[Cu(L) Cl_2]H_2O	[Zn(L) $(H_2O)_2Cl_2$] H_2O	[Cd(L)Cl (H_2O)] Cl.2H_2O	**Assignment**
3424sh	3437sh	3428sh	3387sh	3420sh	3408sh	3440sh	3443sh	3450sh	NH
1696sh	Disappear	disappear	disappear	Disappear	Disappear	Disappear	disappear	Disappear	CONH
1728sh	1730sh	1729w	1730sh	1730sm	1795w	1716sm	1700sm	1700m	C=O
1616s	1619s	1622m	1616 sh	1617m	1620m	1619 m	1617m	1617m	C=N
-------	529w	503w	512w	524w	503w	519w	518w	522w	M-O stretch
-------	427w	427w	492w	473w	427w	415w	460w	420w	M-N stretch
-------	879, 944 Sm	---------	873, 951sm	876, 945sm	872, 955sm	--------	874, 952sm	873, 911sm	Water of coordination

sh = sharp; s = strong; m = medium; sm = small; w = weak

Table S2. Diffused reflectance spectral data of binary metal complexes.

Complex	Band position (cm^{-1})	Transition	Geometry
$Cr[(L)(H_2O)_2Cl_2]Cl.H_2O$	21,834<br>19,841<br>17,301	$^4A_{2g}(F)\rightarrow{}^4T_{1g}(P)$,<br>$^4A_{2g}(F)\rightarrow{}^4T_{2g}(F)$,<br>$^4A_{2g}(F)\rightarrow{}^4T_{2g}(F)$	Octahedral
$[Mn(L)Cl_2]$	21,008<br>18,465<br>15,822	$^4T_{1g}(D)\rightarrow{}^6A_{1g}$&$^4T_{2g}(G)\rightarrow{}^6A_{1g}$,<br>$^6A_{1g}\rightarrow{}^4T_{2g}(D)$	Tetrahedral
$[Fe(L)Cl_2(H_2O)_2]Cl .H_2O$	20,661<br>17,421 & 15,267	$^6A_{1g}\rightarrow T_{2g}(G)$,<br>$^6A_{1g}\rightarrow{}^5T_{1g}$,	Octahedral
$[Co(L)(H_2O)_3Cl]Cl.H_2O$	23,640<br>19,723<br>17,421	$^4T_{1g}\rightarrow{}^4T_g(P)$,<br>$^4T_{1g}\rightarrow{}^4A_{2g}(F)$<br>$^4T_{1g}\rightarrow{}^4T_{2g}(F)$,	Octahedral
$[Ni(L)(H_2O)_2Cl_2]2H_2O$	23,640<br>16,025<br>14,456	$^3A_{2g}$ (F) $\rightarrow{}^3T_{1g}$ (P),<br>$^3A_{2g}$ (F) $\rightarrow{}^3T_{1g}$ (F),	Octahedral
$[Cu(L)Cl_2]H_2O$	23.310<br>19,120	$^2B_{1g}\rightarrow{}^2A_{1g}$<br>$^2B_{1g}\rightarrow{}^2E_g$	Tetrahedral
$[Zn(L)(H_2O)_2Cl_2]H_2O$	23,265<br>17,361<br>16,949	charge transfer(LMCT)<br>$^3T_{1g}(F)\rightarrow{}^3T_{2g}(P)$<br>$^3T_{1g}(F)\rightarrow{}^3A_{1g}$	Octahedral

Table S3. Physiochemical properties of synthesized compounds.

	L	Cr	Mn	Fe	Co	Ni	Cu	Zn	Cd
Molecular weight	222.24 g/mol	381.17 g/mol	348.09 g/mol	385.02 g/mol	370.67 g/mol	387.87 g/mol	356.69 g/mol	394.56 g/mol	388.12 g/mol
Num. heavy atoms	17	22	20	22	22	22	20	22	20
Num. arom. heavy atoms	12	15	15	15	15	15	15	15	15
Fraction Csp3	0.00	0.00	0.00	0.00	0.00	0.00	0.00	0.00	0.00
Num. rotatable bonds	1	1	1	1	1	1	1	1	1
Num. H–bond acceptors	2	3	1	3	4	3	1	3	2
Num. H–bond donors	1	3	1	3	4	3	1	3	2
Molar Refractivity	70.69	88.55	82.45	88.55	85.74	88.55	82.45	88.55	79.65
TPSA	41.46 Å²	63.68 Å²	45.22 Å²	63.68 Å²	72.91 Å²	63.68 Å²	45.22 Å²	63.68 Å²	54.45 Å²

TPSA: Topological polar surface area

Table S4. Lipophilicity and water solubility of synthesized compounds.

	L	Cr	Mn	Fe	Co	Ni	Cu	Zn	Cd
Log Po/w (iLOGP)	1.87	0.00	0.00	0.00	0.00	0.00	0.00	0.00	0.00
Log Po/w (XLOGP3)	2.67	3.00	3.95	3.00	1.73	3.00	3.95	3.00	2.68
Log Po/w (WLOGP)	2.45	3.07	3.20	3.07	2.31	3.07	3.19	3.07	2.44
Log Po/w (MLOGP)	2.33	1.01	2.69	1.01	-0.06	1.01	2.69	1.01	1.59
Log Po/w (SILICOS-IT)	3.27	-1.35	1.43	-1.33	-3.10	-1.31	1.48	-1.27	-0.01
Consensus Log Po/w	2.52	1.14	2.25	1.15	0.18	1.15	2.26	1.16	1.34
Log S (ESOL)	-3.36	-4.53	-4.98	-4.56	-3.67	-4.57	-5.03	-4.61	-4.42
Solubility	9.78e-02 mg/ml; 4.40e-04 mol/l	1.12e-02 mg/ml; 2.94e-05 mol/l	3.58e-03 mg/ml; 1.06e-05 mol/l	1.07e-02 mg/ml; 2.78e-05 mol/l	7.99e-02 mg/ml; 2.15e-04 mol/l	1.04e-02 mg/ml; 2.67e-05 mol/l	3.34e-03 mg/ml; 9.35e-06 mol/l	9.58e-03 mg/ml; 2.43e-05 mol/l	1.46e-02 mg/ml; 3.77e-05 mol/l
Class	Soluble	Moderately soluble	Moderately soluble	Moderately soluble	Soluble	Moderately soluble	Moderately soluble	Moderately soluble	Moderately soluble
Log S (Ali)	-3.19	-4.00	-4.60	-4.00	-2.88	-4.00	-4.60	-4.00	-3.48
Solubility	1.43e-01 mg/ml ; 6.42e-04 mol/l	3.80e-02 mg/ml ; 9.96e-05 mol/l	8.75e-03 mg/ml ; 2.51e-05 mol/l	3.84e-03 mg/ml ; 9.96e-05 mol/l	4.91e-01 mg/ml ; 1.33e-03 mol/l	3.86e-02 mg/ml ; 9.96e-05 mol/l	8.97e-03 mg/ml ; 2.51e-05 mol/l	3.93e-02 mg/ml ; 9.96e-05 mol/l	1.30e-01 mg/ml ; 3.34e-04 mol/l
Class	Soluble	Moderately soluble	Moderately soluble	Moderately soluble	Soluble	Moderately soluble	Moderately soluble	Moderately soluble	Soluble
Log S (SILICOS-IT)	-5.45	-7.38	-7.29	-7.39	-7.39	-7.40	-7.31	-7.42	-6.90
Solubility	7.96e-04 mg/ml; 3.58e-06 mol/l	1.59e-05 mg/ml; 4.17e-08 mol/l	1.78e-05 mg/ml; 5.12e-08 mol/l	1.56e-05 mg/ml; 4.06e-08 mol/l	5.18e-05 mg/m; 1.40e-07 mol/l	1.55e-05 mg/ml; 4.00e-08 mol/l	1.73e-05 mg/ml; 4.85e-08 mol/l	1.51e-05 mg/ml; 3.85e-08 mol/l	4.86e-05 mg/ml; 1.25e-07 mol/l
Class	Moderately soluble	Poorly soluble	Poorly soluble	Poorly soluble	Poorly soluble	Poorly soluble	Poorly soluble	Poorly soluble	Poorly soluble

Table S5. Biological activity of Schiff base ligand and its metal complexes with Gram positive and Gram negative bacteria and with fungi.

**Sample**	**Inhibition zone diameter (mm / mg sample)**					
	Gram positive		***Gram negative***		***Fungi***	
	Streptococcus pneumoniae	***Bacillus Subtilis***	***Pseudomonas aeruginosa***	***Escherichia coli***	***Aspergillus fumigatus***	***Candida albicans***
L	10.90	12.90	NA	11.60	NA	10.80
$Cr[(L)(H_2O)_2Cl_2]Cl.H_2O$	18.70	15.40	11.70	10.30	14.90	16.10
$[Mn(L)Cl_2]$	11.60	12.60	11.90	10.10	NA	10.50
$[Fe(L)Cl_2(H_2O)_2]Cl.H_2O$	12.60	13.20	10.10	10.90	NA	9.30
$[Co(L)(H_2O)_3Cl]Cl.H_2O$	14.10	12.80	11.40	10.10	12.70	15.20
$[Ni(L)(H_2O)_2Cl_2]2H_2O$	19.60	12.90	9.70	8.90	15.40	12.90
$[Cu(L)Cl)_2]H_2O$	17.30	13.30	10.30	10.90	NA	11.50
$[Zn(L)(H_2O)_2Cl_2]H_2O$	13.40	11.60	NA	9.90	9.80	14.60
$[Cd(L)Cl(H_2O)]Cl.2H_2O$	17.90	12.70	11.30	9.90	19.60	14.80
Amikacin	9	6	7	6	-------	---------
Ketokonazole	--------	--------	----------	---------	9	9

Table S6. Anti-breast cancer activity of Schiff base ligand and its metal complexes.

Surviving fraction	0	5	12.5	25	50	IC_{50} µg/ml
Ligand (L)	1	0.71	0.55	0.31	0.30	15.20
$Cr[(L)(H_2O)_2Cl_2]Cl.H_2O$	1	0.90	0.75	0.38	0.29	21.00
$[Mn(L)Cl_2]$	1	0.87	0.66	0.32	0.27	18.40
$[Fe(L)Cl_2(H_2O)_2]Cl.H_2O$	1	0.86	0.66	0.36	0.39	19.10
$[Co(L)(H_2O)_3Cl]Cl.H_2O$	1	0.90	0.67	0.45	0.41	22.30
$[Ni(L)(H_2O)_2Cl_2]2H_2O$	1	0.91	0.63	0.33	0.31	18.10
$[Cu(L)Cl_2]H_2O$	1	0.79	0.50	0.28	0.27	15.00
$[Zn (L)(H_2O)_2Cl_2]H_2O$	1	0.82	0.52	0.26	0.27	13.80
$[Cd(L)Cl(H_2O)]Cl.2H_2O$	1	0.81	0.48	0.27	0.26	12.00

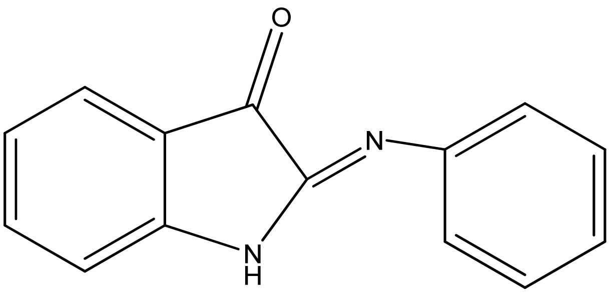

(*Z*)-2-(phenylimino)indolin-3-one

Figure S1. Structure of the Schiff base ligand (L).

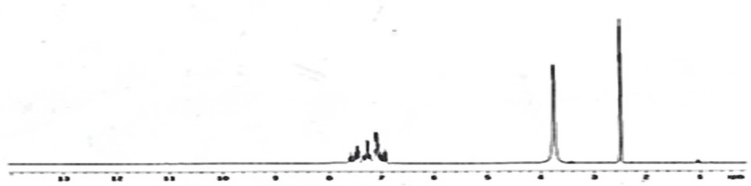

Figure S2. ^{1}H NMR spectrum of Schiff base ligand (L).

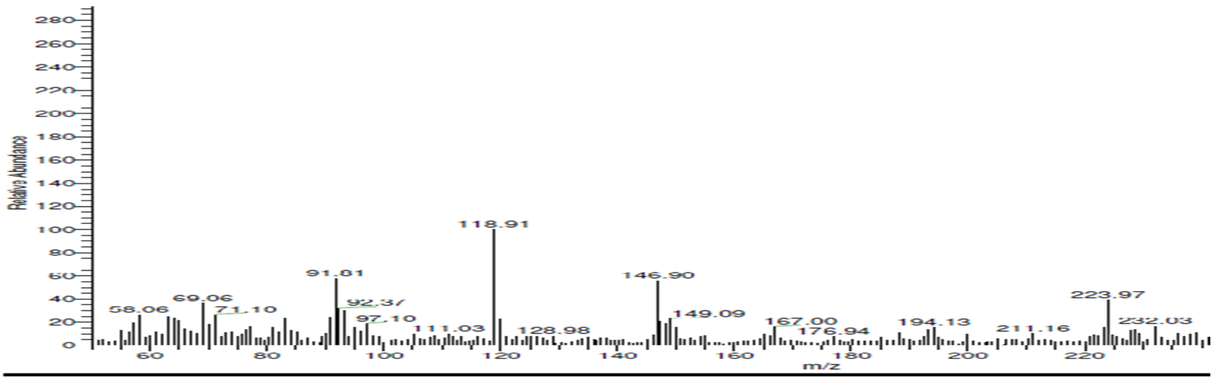

Figure S3. Mass spectrum of Schiff base ligand (L).

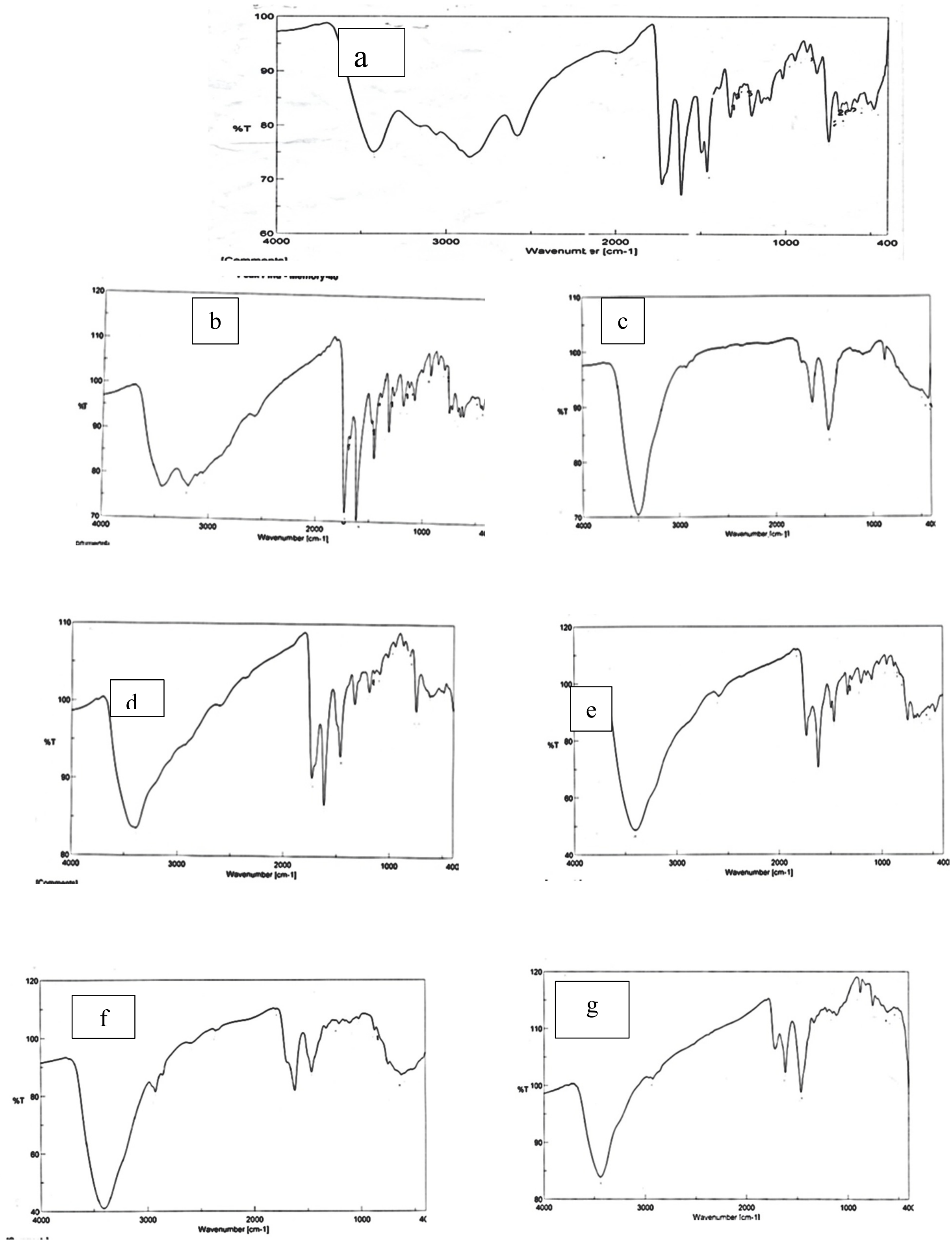

a
b
c
d
e
f
g
%T
Wavenumber [cm-1]
4000
3000
2000
1000
400

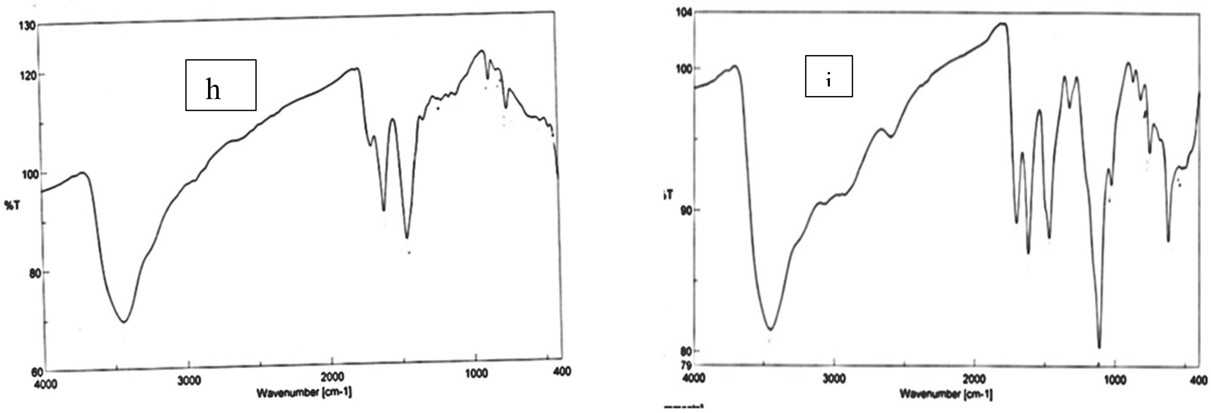

Figure S4. IR spectra of (a) Schiff base ligand (L), (b) Cr(III), (c) Mn(II), (d) Fe(III), (e) Co(II), (f) Ni(II), (g) Cu(II), (h) Zn(II), and (i) Cd(II) complexes.

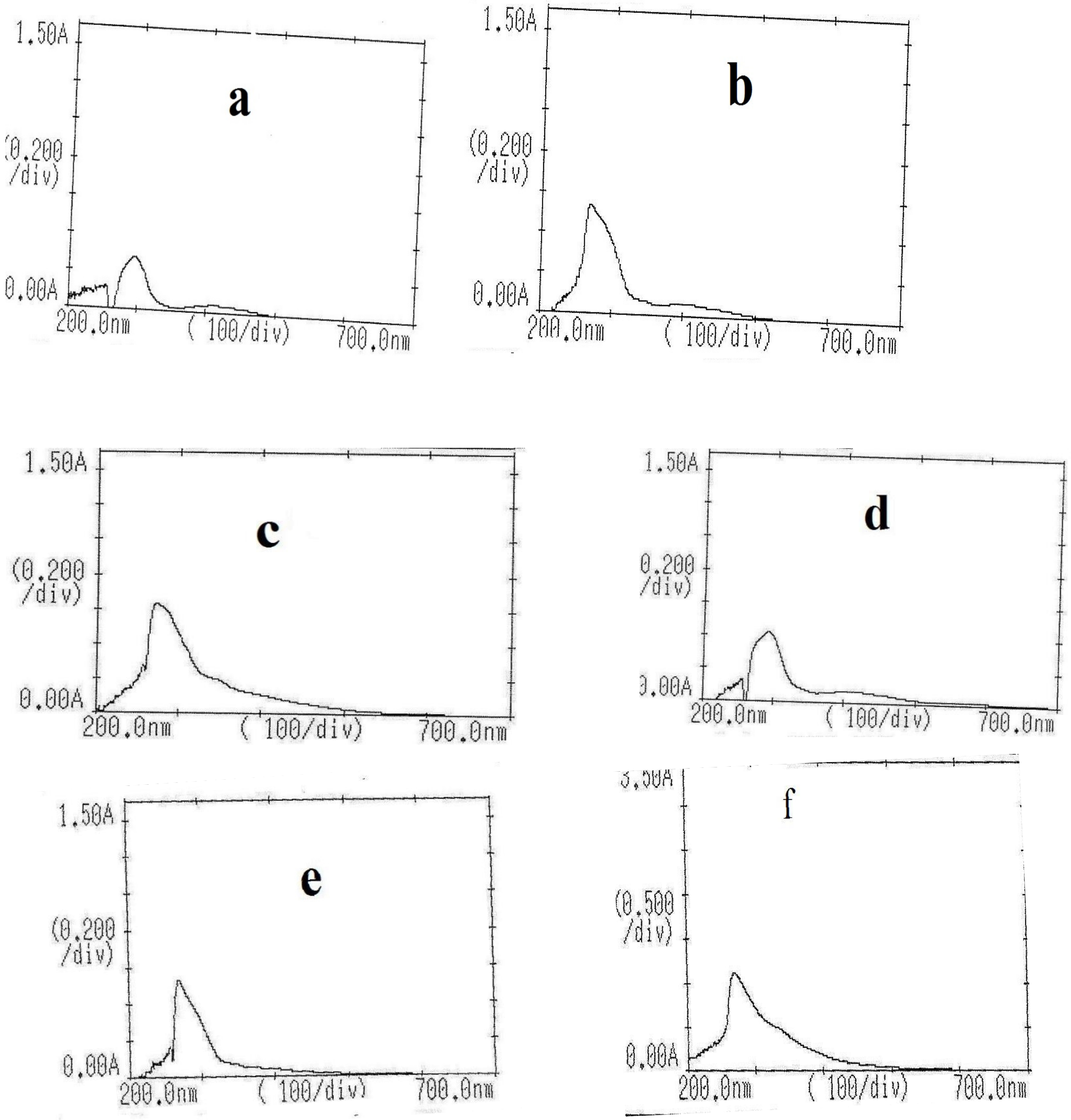
a
b
c
d
e
f
1.50A
(0.200 /div)
0.00A
200.0nm
(100/div)
700.0nm
3.50A
(0.500 /div)

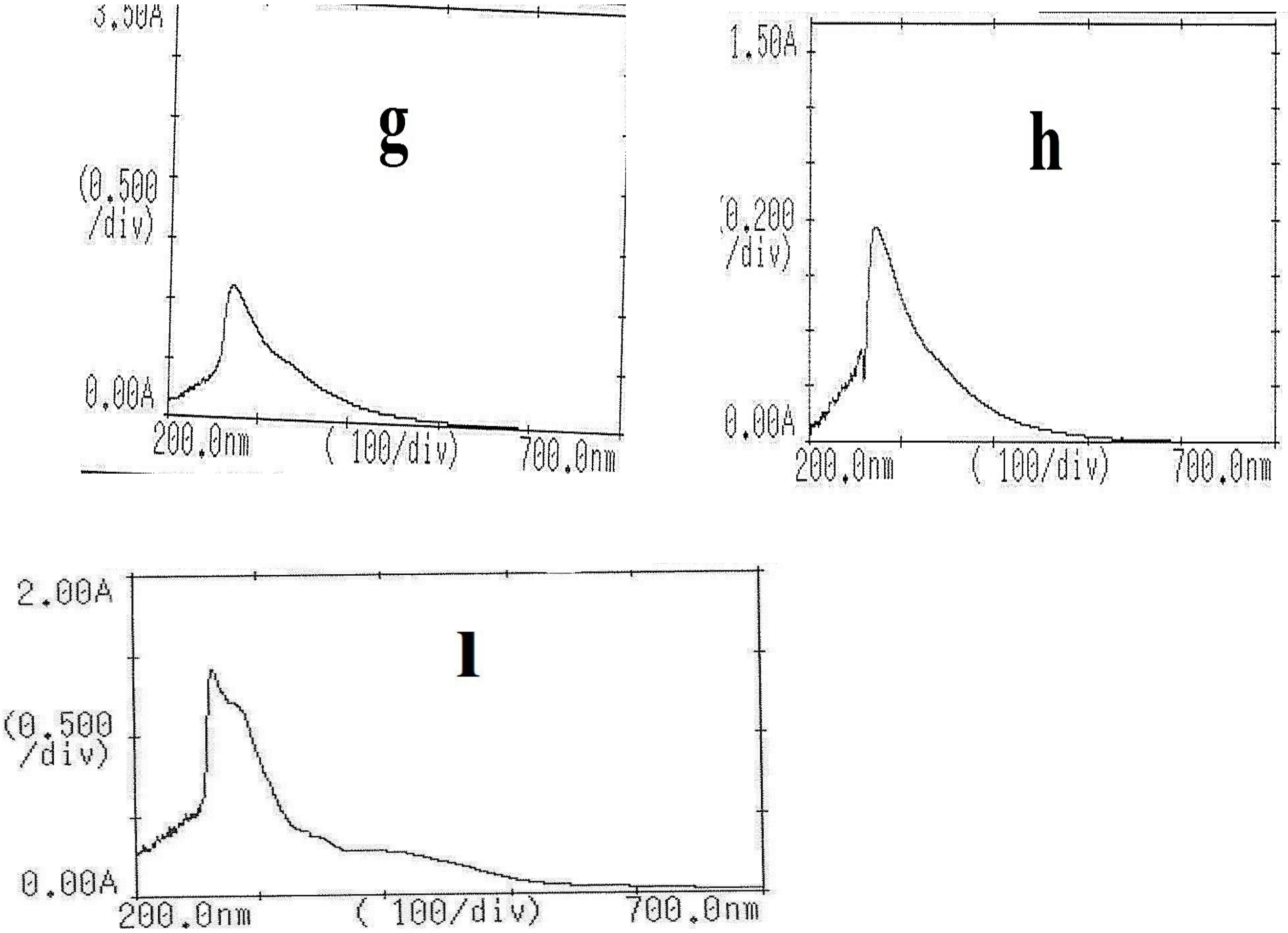

Figure S5. The UV-Vis absorption spectra of (l) Schiff base ligand (L), (a) Cr(III), (b) Mn(II), (c) Fe(III), (d) Co(II), (e) Ni(II), (f) Cu(II), (g) Zn(II), and (h) Cd(II) complexes.

a

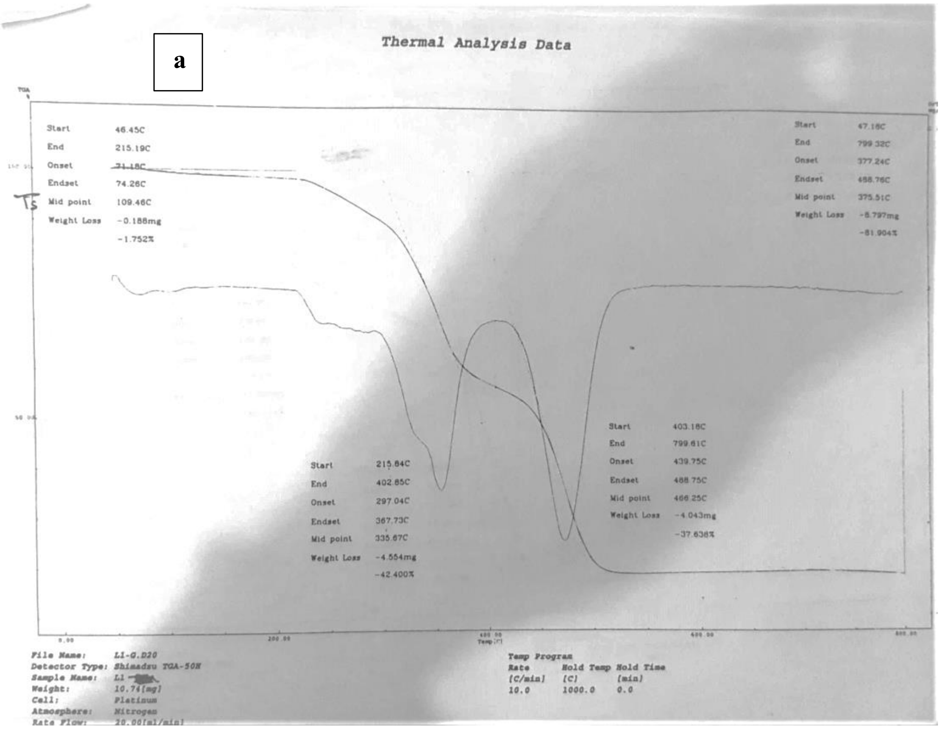
Thermal Analysis Data
Start 46.45C
End 215.19C
Onset 71.18C
Endset 74.26C
Mid point 109.46C
Weight Loss -0.188mg
-1.752%
Start 47.16C
End 799.32C
Onset 377.24C
Endset 488.76C
Mid point 375.51C
Weight Loss -8.797mg
-81.904%
Start 403.18C
End 799.61C
Onset 439.75C
Endset 488.75C
Mid point 466.25C
Weight Loss -4.043mg
-37.636%
Start 215.84C
End 402.85C
Onset 297.04C
Endset 367.73C
Mid point 335.67C
Weight Loss -4.554mg
-42.400%
File Name: LI-G.D20
Detector Type: Shimadzu TGA-50H
Sample Name: LI [illegible]
Weight: 10.74[mg]
Cell: Platinum
Atmosphere: Nitrogen
Rate Flow: 20.00[ml/min]
Temp Program
Rate [C/min] 10.0
Hold Temp [C] 1000.0
Hold Time [min] 0.0

b

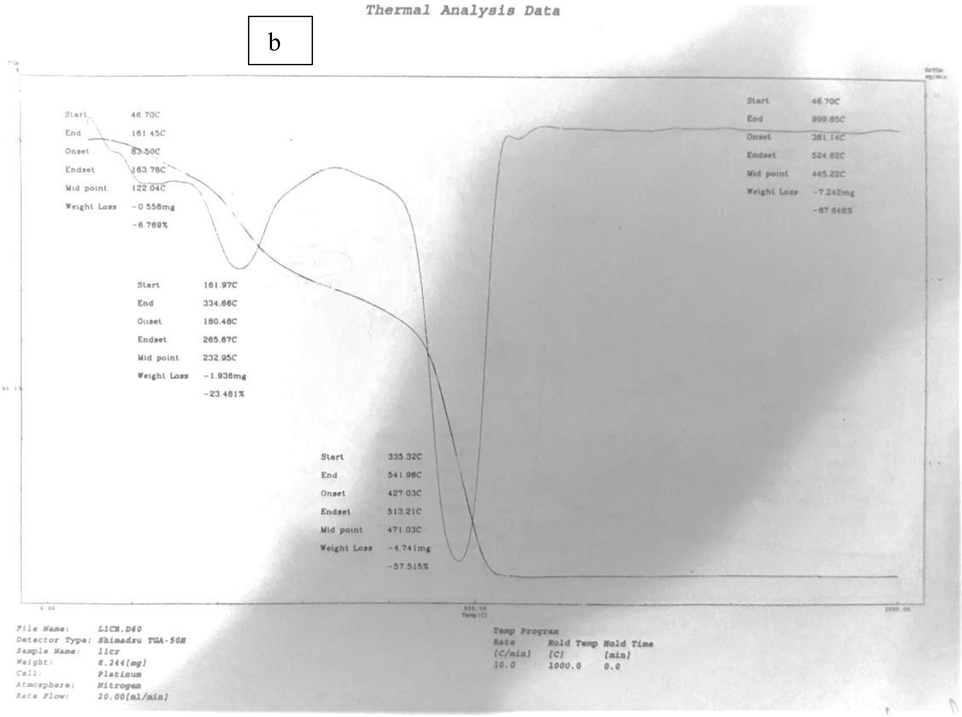
Thermal Analysis Data
Start 46.70C
End 161.45C
Onset 83.50C
Endset 163.78C
Mid point 122.04C
Weight Loss -0.558mg
-6.769%
Start 46.70C
End 999.85C
Onset 361.14C
Endset 524.82C
Mid point 445.22C
Weight Loss -7.242mg
-87.848%
Start 161.97C
End 334.86C
Onset 180.48C
Endset 265.67C
Mid point 232.95C
Weight Loss -1.936mg
-23.481%
Start 335.32C
End 541.96C
Onset 427.03C
Endset 513.21C
Mid point 471.03C
Weight Loss -4.741mg
-57.515%
File Name: L1CR.D60
Detector Type: Shimadzu TGA-50H
Sample Name: l1cr
Weight: 8.244[mg]
Cell: Platinum
Atmosphere: Nitrogen
Rate Flow: 20.00[ml/min]
Temp Program
Rate [C/min] 10.0
Hold Temp [C] 1000.0
Hold Time [min] 0.0

c

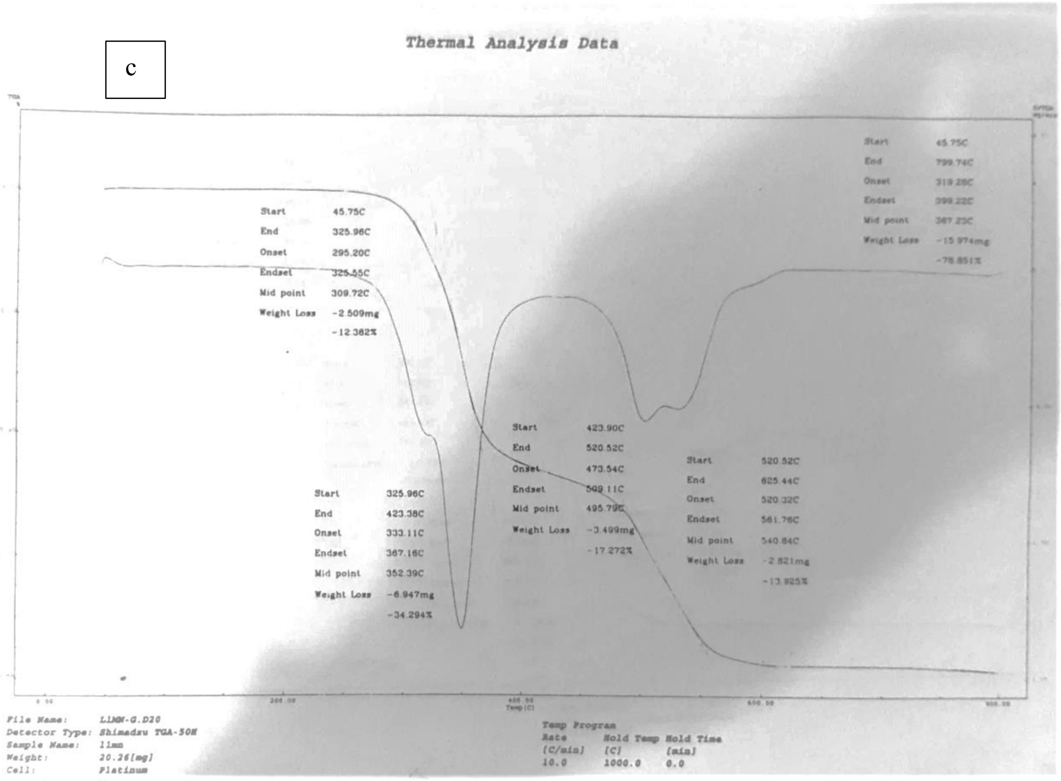
Thermal Analysis Data
Start 45.75C
End 325.96C
Onset 295.20C
Endset 325.55C
Mid point 309.72C
Weight Loss -2.509mg
-12.382%
Start 325.96C
End 423.38C
Onset 333.11C
Endset 367.16C
Mid point 352.39C
Weight Loss -6.947mg
-34.294%
Start 423.90C
End 520.52C
Onset 473.54C
Endset 509.11C
Mid point 495.79C
Weight Loss -3.499mg
-17.272%
Start 520.52C
End 625.44C
Onset 520.32C
Endset 561.76C
Mid point 540.84C
Weight Loss -2.821mg
-13.925%
Start 45.75C
End 799.74C
Onset 319.28C
Endset 399.22C
Mid point 367.23C
Weight Loss -15.974mg
-78.851%
File Name: LIMN-G.D20
Detector Type: Shimadzu TGA-50H
Sample Name: llmn
Weight: 20.26[mg]
Cell: Platinum
Temp Program
Rate Hold Temp Hold Time
[C/min] [C] [min]
10.0 1000.0 0.0

d

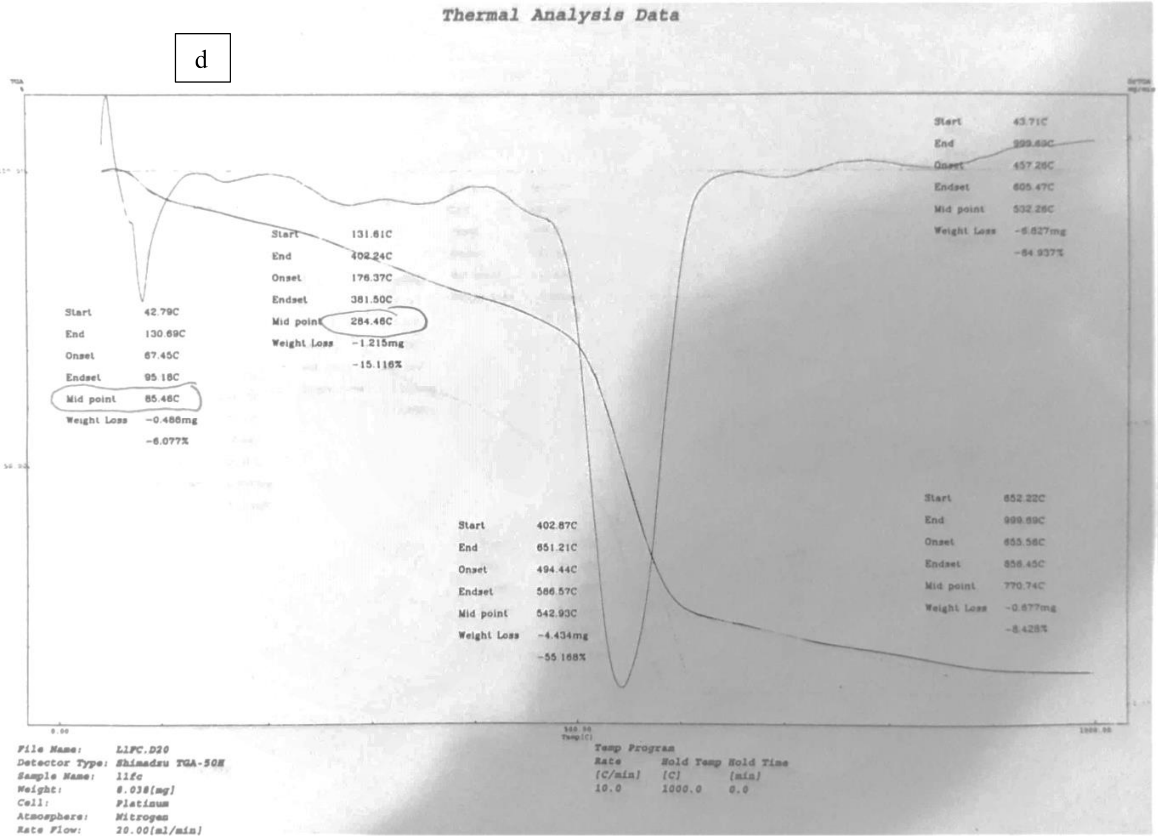
Thermal Analysis Data
Start 42.79C
End 130.69C
Onset 67.45C
Endset 95.18C
Mid point 85.46C
Weight Loss -0.488mg
-6.077%
Start 131.61C
End 402.24C
Onset 176.37C
Endset 381.50C
Mid point 284.46C
Weight Loss -1.215mg
-15.116%
Start 402.87C
End 651.21C
Onset 494.44C
Endset 586.57C
Mid point 542.93C
Weight Loss -4.434mg
-55.168%
Start 43.71C
End 999.69C
Onset 457.26C
Endset 605.47C
Mid point 532.26C
Weight Loss -6.827mg
-84.937%
Start 652.22C
End 999.69C
Onset 655.56C
Endset 856.45C
Mid point 770.74C
Weight Loss -0.677mg
-8.428%
File Name: LLFC.D20
Detector Type: Shimadzu TGA-50H
Sample Name: llfc
Weight: 8.038[mg]
Cell: Platinum
Atmosphere: Nitrogen
Rate Flow: 20.00[ml/min]
Temp Program
Rate Hold Temp Hold Time
[C/min] [C] [min]
10.0 1000.0 0.0

e

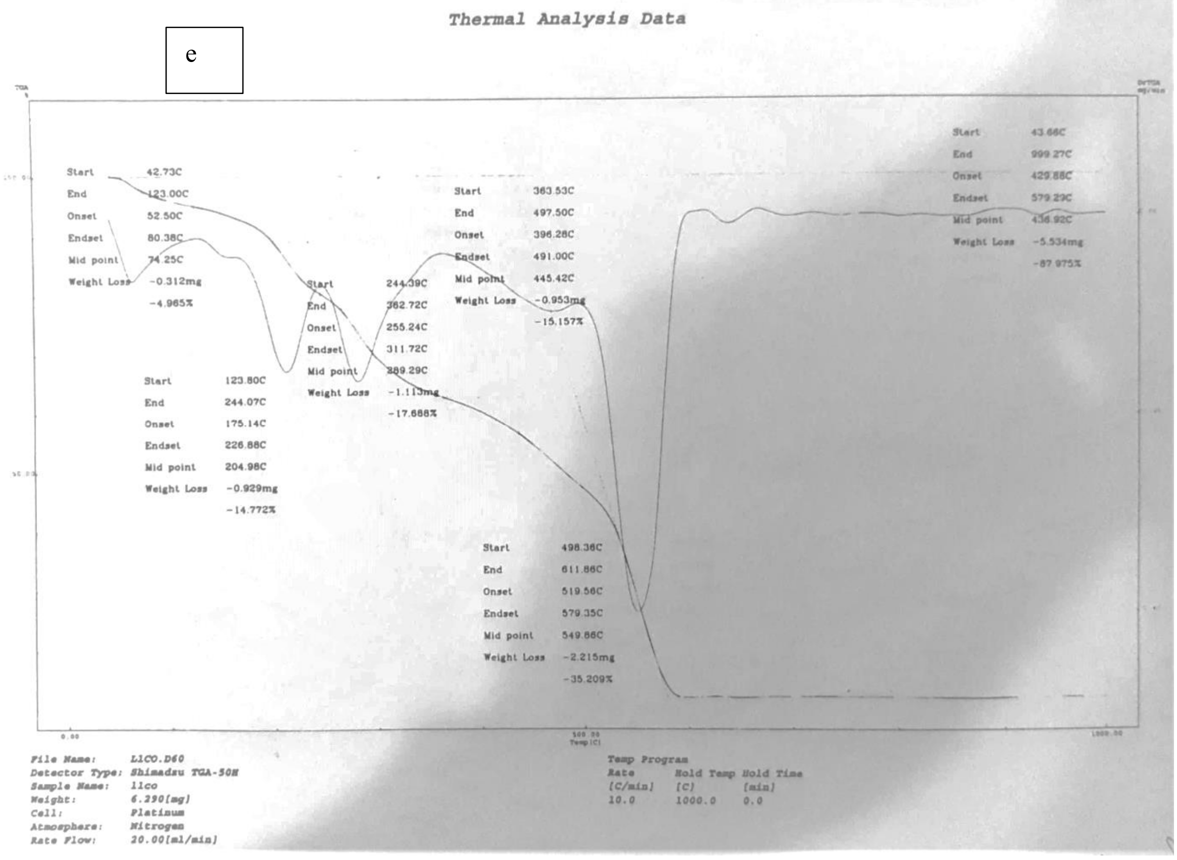

Thermal Analysis Data
TGA
Start 42.73C
End 123.00C
Onset 52.50C
Endset 80.38C
Mid point 74.25C
Weight Loss -0.312mg
-4.965%
Start 123.80C
End 244.07C
Onset 175.14C
Endset 226.88C
Mid point 204.98C
Weight Loss -0.929mg
-14.772%
Start 244.39C
End 382.72C
Onset 255.24C
Endset 311.72C
Mid point 289.29C
Weight Loss -1.113mg
-17.688%
Start 363.53C
End 497.50C
Onset 396.28C
Endset 491.00C
Mid point 445.42C
Weight Loss -0.953mg
-15.157%
Start 498.36C
End 611.86C
Onset 519.56C
Endset 579.35C
Mid point 549.86C
Weight Loss -2.215mg
-35.209%
Start 43.66C
End 999.27C
Onset 429.88C
Endset 579.29C
Mid point 436.92C
Weight Loss -5.534mg
-87.975%
0.00
500.00
Temp [C]
1000.00
File Name: LICO.D60
Detector Type: Shimadzu TGA-50H
Sample Name: llco
Weight: 6.290[mg]
Cell: Platinum
Atmosphere: Nitrogen
Rate Flow: 20.00[ml/min]
Temp Program
Rate Hold Temp Hold Time
[C/min] [C] [min]
10.0 1000.0 0.0

f

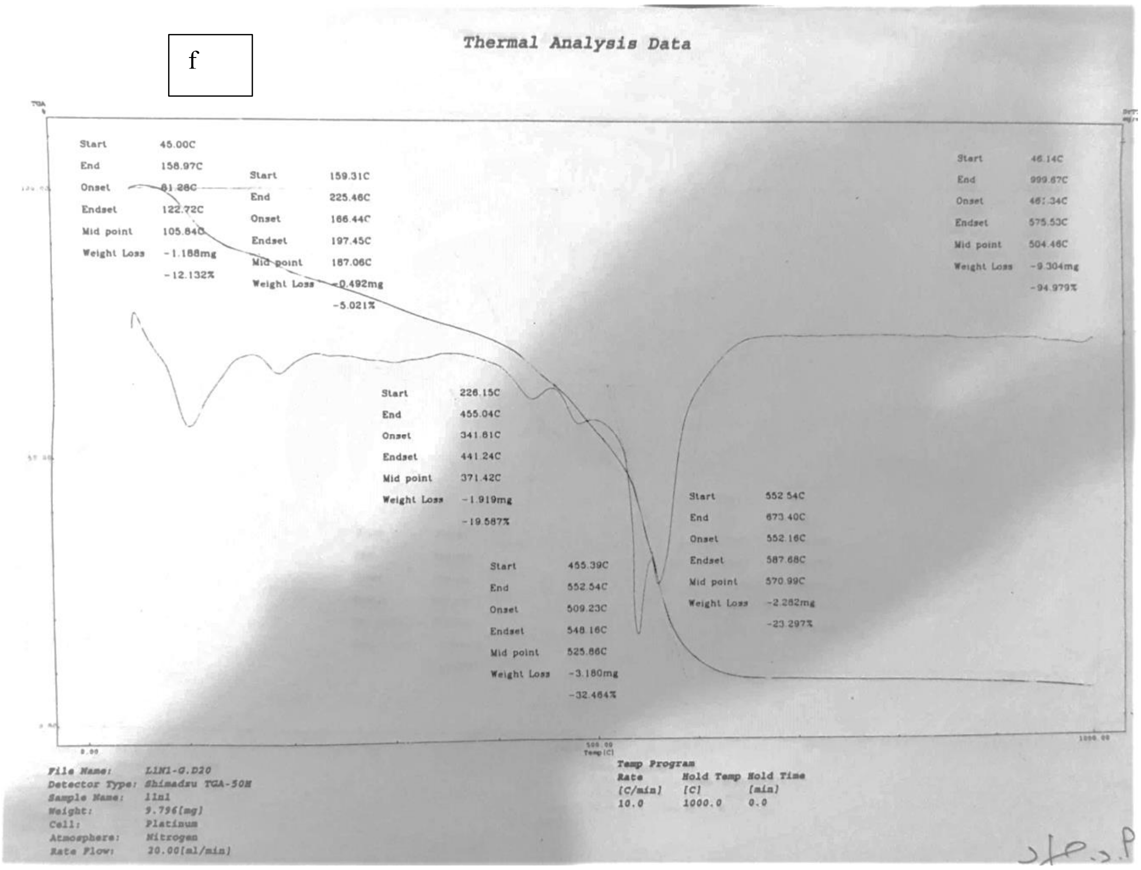

Thermal Analysis Data
TGA
Start 45.00C
End 158.97C
Onset 81.28C
Endset 122.72C
Mid point 105.84C
Weight Loss -1.188mg
-12.132%
Start 159.31C
End 225.46C
Onset 166.44C
Endset 197.45C
Mid point 187.06C
Weight Loss -0.492mg
-5.021%
Start 226.15C
End 455.04C
Onset 341.61C
Endset 441.24C
Mid point 371.42C
Weight Loss -1.919mg
-19.587%
Start 455.39C
End 552.54C
Onset 509.23C
Endset 548.16C
Mid point 525.86C
Weight Loss -3.180mg
-32.464%
Start 552.54C
End 673.40C
Onset 552.16C
Endset 587.68C
Mid point 570.99C
Weight Loss -2.282mg
-23.297%
Start 46.14C
End 999.67C
Onset 461.34C
Endset 575.53C
Mid point 504.46C
Weight Loss -9.304mg
-94.979%
0.00
500.00
Temp [C]
1000.00
File Name: LIN1-G.D20
Detector Type: Shimadzu TGA-50H
Sample Name: lin1
Weight: 9.796[mg]
Cell: Platinum
Atmosphere: Nitrogen
Rate Flow: 20.00[ml/min]
Temp Program
Rate Hold Temp Hold Time
[C/min] [C] [min]
10.0 1000.0 0.0

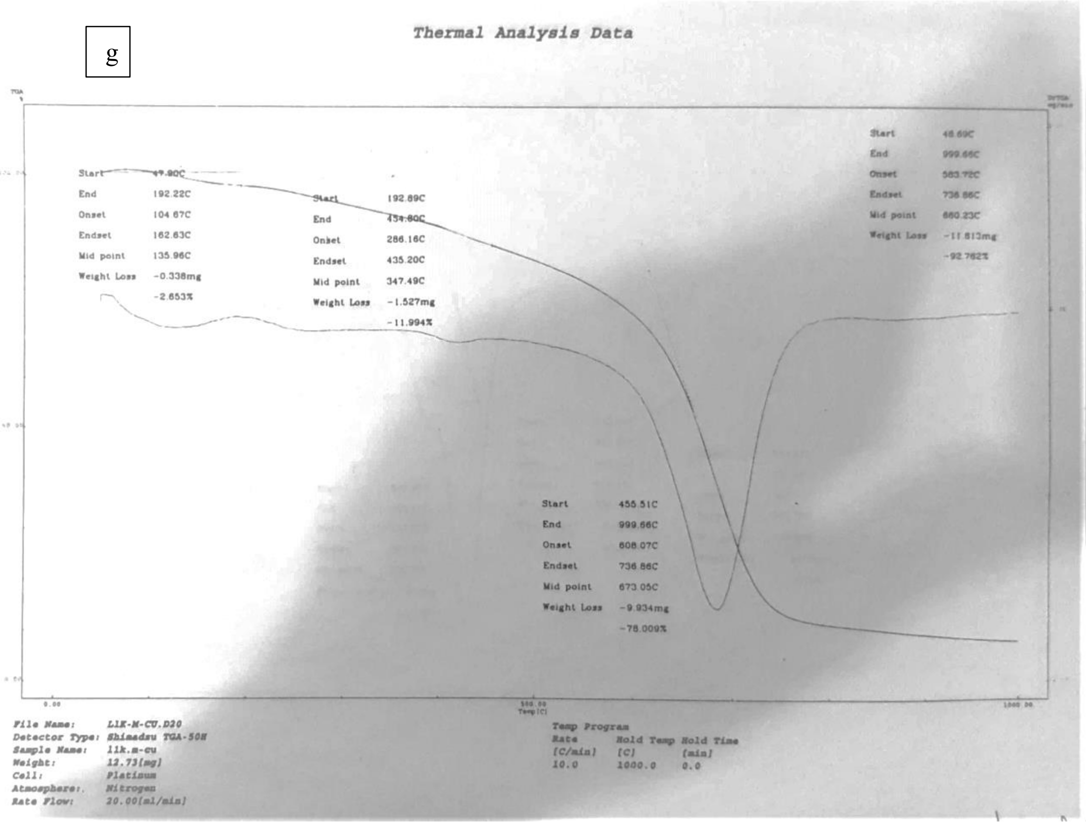
g
Thermal Analysis Data
Start 47.90C
End 192.22C
Onset 104.67C
Endset 162.63C
Mid point 135.96C
Weight Loss -0.338mg
-2.653%
Start 192.89C
End 454.80C
Onset 286.16C
Endset 435.20C
Mid point 347.49C
Weight Loss -1.527mg
-11.994%
Start 46.69C
End 999.66C
Onset 583.72C
Endset 736.86C
Mid point 660.23C
Weight Loss -11.813mg
-92.762%
Start 455.51C
End 999.66C
Onset 608.07C
Endset 736.86C
Mid point 673.05C
Weight Loss -9.934mg
-78.009%
File Name: L1K-M-CU.D20
Detector Type: Shimadzu TGA-50H
Sample Name: l1k.m-cu
Weight: 12.73[mg]
Cell: Platinum
Atmosphere: Nitrogen
Rate Flow: 20.00[ml/min]
Temp Program
Rate Hold Temp Hold Time
[C/min] [C] [min]
10.0 1000.0 0.0

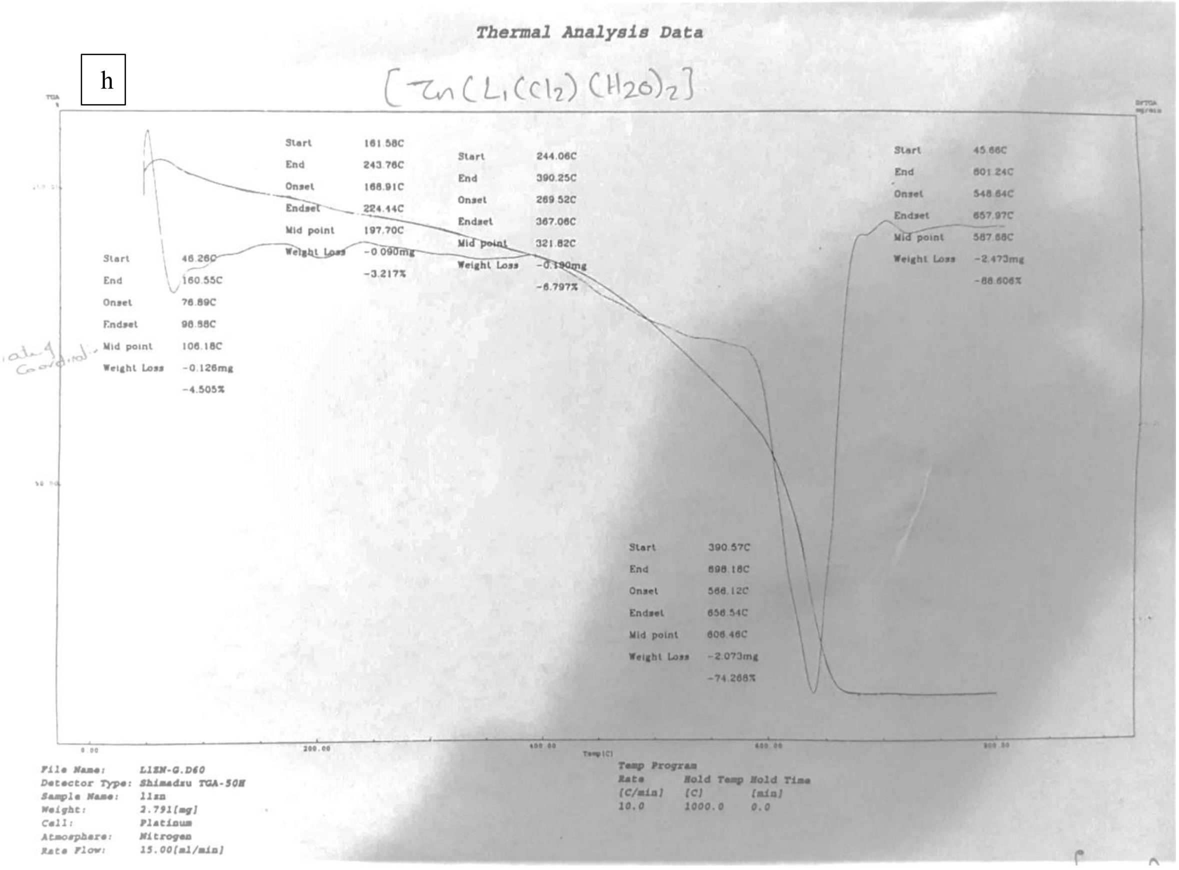
h
Thermal Analysis Data
[Zn(L1(Cl2)(H2O)2]
Start 161.58C
End 243.76C
Onset 168.91C
Endset 224.44C
Mid point 197.70C
Weight Loss -0.090mg
-3.217%
Start 244.06C
End 390.25C
Onset 269.52C
Endset 367.06C
Mid point 321.82C
Weight Loss -0.190mg
-6.797%
Start 45.66C
End 801.24C
Onset 548.64C
Endset 657.97C
Mid point 587.88C
Weight Loss -2.473mg
-88.606%
Start 46.26C
End 160.55C
Onset 76.89C
Endset 96.88C
Mid point 106.18C
Weight Loss -0.126mg
-4.505%
Start 390.57C
End 898.18C
Onset 566.12C
Endset 656.54C
Mid point 606.46C
Weight Loss -2.073mg
-74.268%
File Name: L1ZN-G.D60
Detector Type: Shimadzu TGA-50H
Sample Name: l1zn
Weight: 2.791[mg]
Cell: Platinum
Atmosphere: Nitrogen
Rate Flow: 15.00[ml/min]
Temp Program
Rate Hold Temp Hold Time
[C/min] [C] [min]
10.0 1000.0 0.0

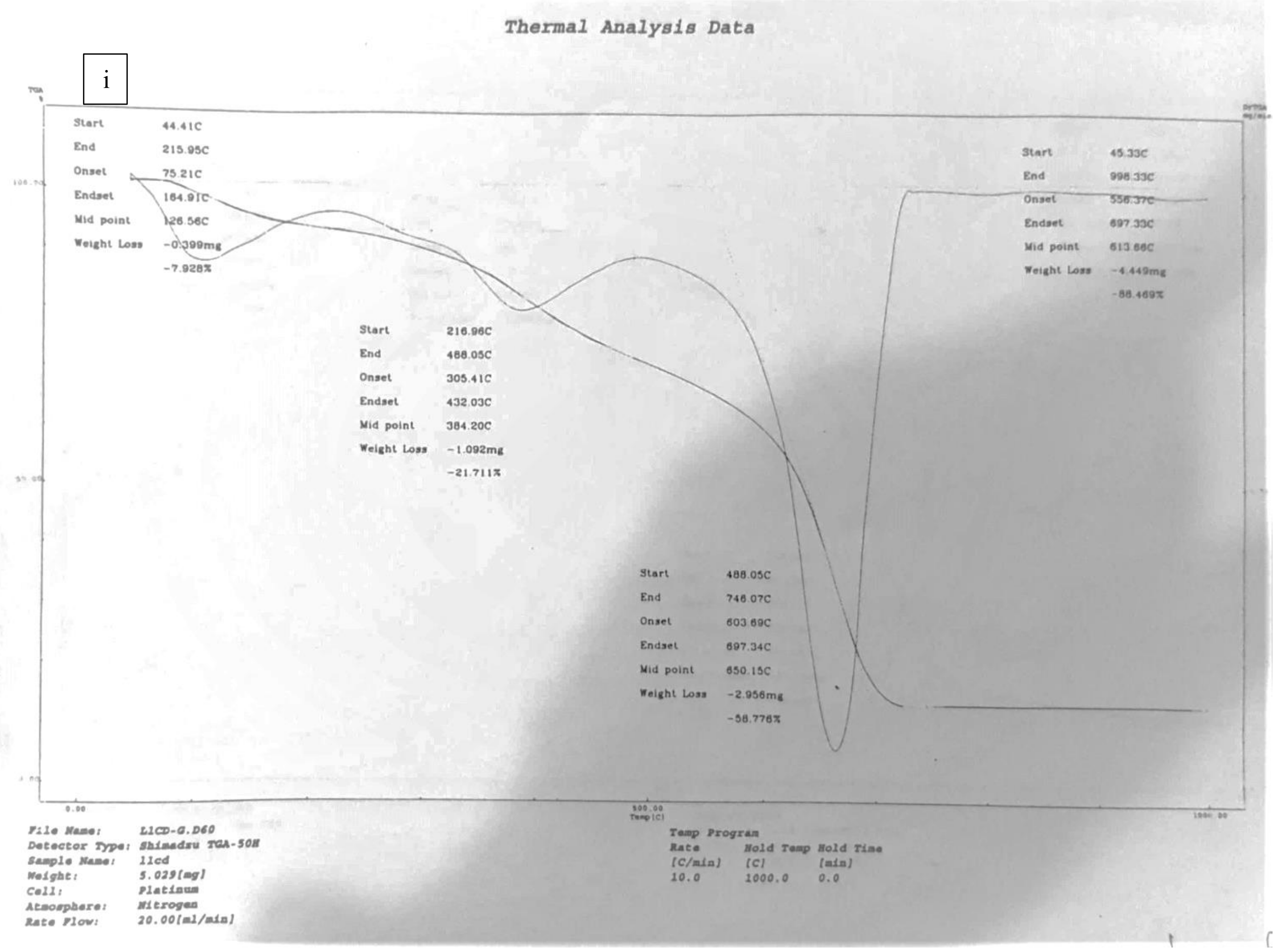

Figure S6. Thermal analysis (TG and DTG) of (a) Schiff base ligand (L), (b) Cr(III), (c) Mn(II), (d) Fe(III), (e) Co(II), (f) Ni(II), (g) Cu(II), (h) Zn(II), and (i) Cd(II) complexes.

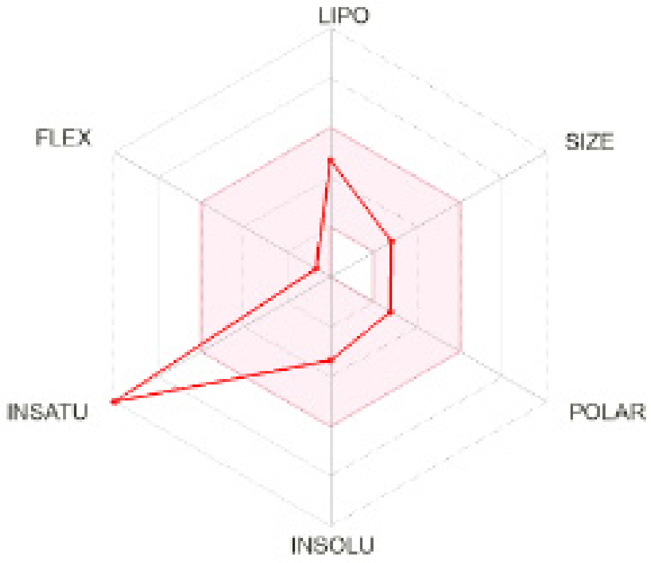

Figure S7. Bioavailability radar chart of the ligand and its metal complexes.
[Pink area in plotted graph represents a favorable set of properties for excellent oral bioavailability. LIPO (lipophilicity), XLOGP between −0.7 and + 5.0, SIZE (molecular weight and range = from 150 to 500 g/mol), POLAR (polarity), TPS (20 and 130 A2), INSOLU (solubility), LogS not higher than six, INSATU (saturation), FLEX (flexibility), and no more than eight rot were able to bond].

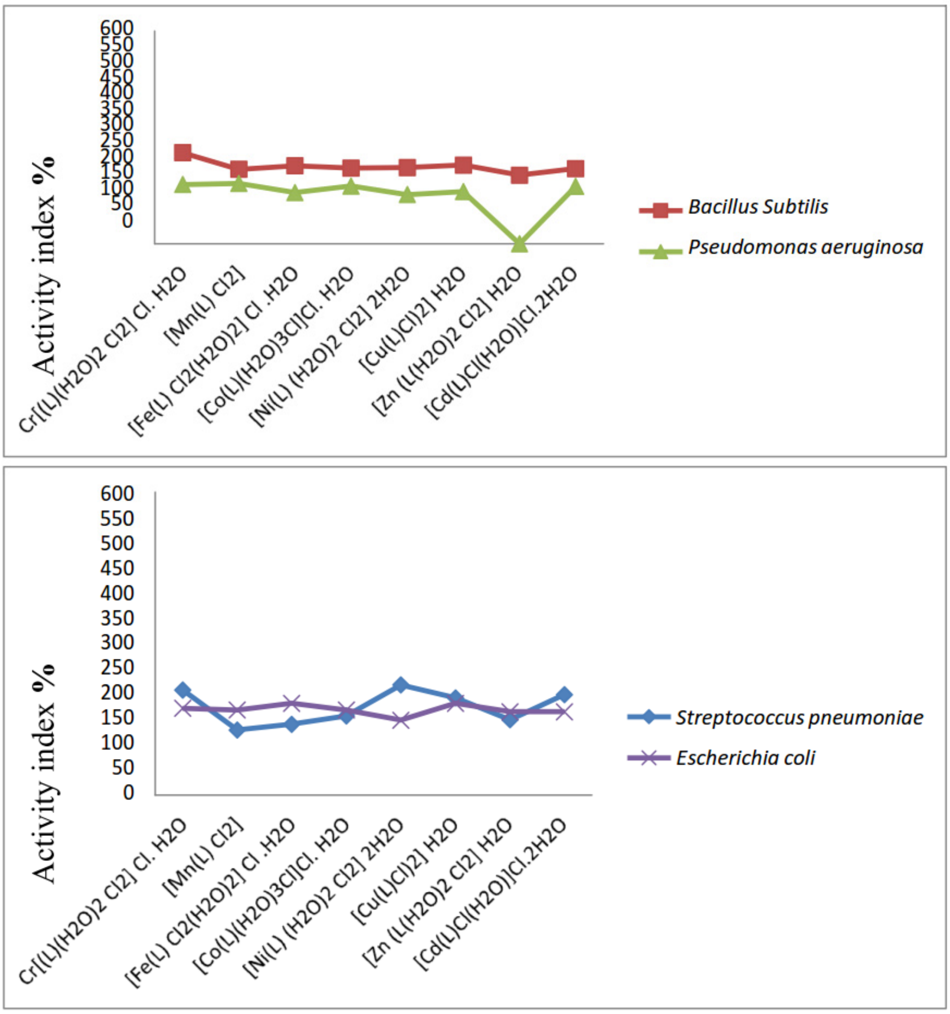

Figure S8. Activity index of the prepared Schiff base complexes.
